# How muscle stiffness affects human body model behavior

**DOI:** 10.1186/s12938-021-00876-6

**Published:** 2021-06-02

**Authors:** Niclas Trube, Werner Riedel, Matthias Boljen

**Affiliations:** Fraunhofer-Institute for High-Speed Dynamics, Ernst-Mach-Institut, EMI, Ernst-Zermelo-Straße 4, 79104 Freiburg, Germany

**Keywords:** Muscle stiffness, Finite element method, FEM, THUMS, Version 5, Human body model, HBM, Frontal impact, Muscle model, Active human body model, AHBM, Occupant safety, Solid muscle elements, Car crash, Numerical, Simulation

## Abstract

**Background:**

Active human body models (AHBM) consider musculoskeletal movement and joint stiffness via active muscle truss elements in the finite element (FE) codes in dynamic application. In the latest models, such as THUMS™ Version 5, nearly all human muscle groups are modeled in form of one-dimensional truss elements connecting each joint. While a lot of work has been done to improve the active and passive behavior of this 1D muscle system in the past, the volumetric muscle system of THUMS was modeled in a much more simplified way based on Post Mortem Human Subject (PMHS) test data. The stiffness changing effect of isometric contraction was hardly considered for the volumetric muscle system of whole human body models so far. While previous works considered this aspect for single muscles, the effect of a change in stiffness due to isometric contraction of volumetric muscles on the AHBM behavior and computation time is yet unknown.

**Methods:**

In this study, a simplified frontal impact using the THUMS Version 5 AM50 occupant model was simulated. Key parameters to regulate muscle tissue stiffness of solid elements in THUMS were identified for the material model MAT_SIMPLIFIED_FOAM and different stiffness states were predefined for the buttock and thigh.

**Results:**

During frontal crash, changes in muscle stiffness had an effect on the overall AHBM behavior including expected injury outcome. Changes in muscle stiffness for the thigh and pelvis, as well as for the entire human body model and for strain-rate-dependent stiffness definitions based on literature data had no significant effect on the computation time.

**Discussion:**

Kinematics, peak impact force and stiffness changes were in general compliance with the literature data. However, different experimental setups had to be considered for comparison, as this topic has not been fully investigated experimentally in automotive applications in the past. Therefore, this study has limitations regarding validation of the frontal impact results.

**Conclusion:**

Variations of default THUMS material model parameters allow an efficient change in stiffness of volumetric muscles for whole AHBM applications. The computation time is unaffected by altering muscle stiffness using the method suggested in this work. Due to a lack of validation data, the results of this work can only be validated with certain limitations. In future works, the default material models of THUMS could be replaced with recently published models to achieve a possibly more biofidelic muscle behavior, which would even allow a functional dependency of the 1D and 3D muscle systems. However, the effect on calculation time and model stability of these models is yet unknown and should be considered in future studies for efficient AHBM applications.

## Core statements

1. The effect of isometric contraction of lower extremity muscles on occupant safety was analyzed by defining different stiffness states for the volumetric muscle elements of THUMS Version 5. Altering the muscle stiffness influenced the overall active human body model (AHBM) behavior during frontal crashes, as well as the predicted injury outcome.
2. Altering the muscle material stiffness via the scaling factor of the ordinate value (SFO) of the load curve referenced in all muscle and soft tissues of THUMS in the LS-DYNA material model MAT_SIMPLIFIED_FOAM has no significant impact on the computation time.
3. Stiffness parameters of the human body model THUMS™ Version 5 are identified, varied and compared to experimental literature data for verification. Results are in general correlation, but verification by precise numerical simulations of the experimental setups should be followed in future studies.
4. Based on strain-dependent injury prediction, the injury risk for muscle and soft tissues during frontal impact is reduced with higher muscle stiffness. Further, based on contact force evaluation, increasing muscle stiffness leads to higher probability of hip fracture or dislocation and to lower probability of knee injuries. Higher risk with increasing muscle stiffness was also found for effective plastic strain evaluation, while first principal strain results rather show an arbitrary influence of muscle stiffness on the injury risk of cortical and spongy bones. Further studies are necessary to investigate the exact influence of muscle stiffness on bone injury risk in detail.

## Background

This work shall provide major explications and additional information of a study previously presented by the authors [1].

### Development of active human body models

**Fig. 1:**
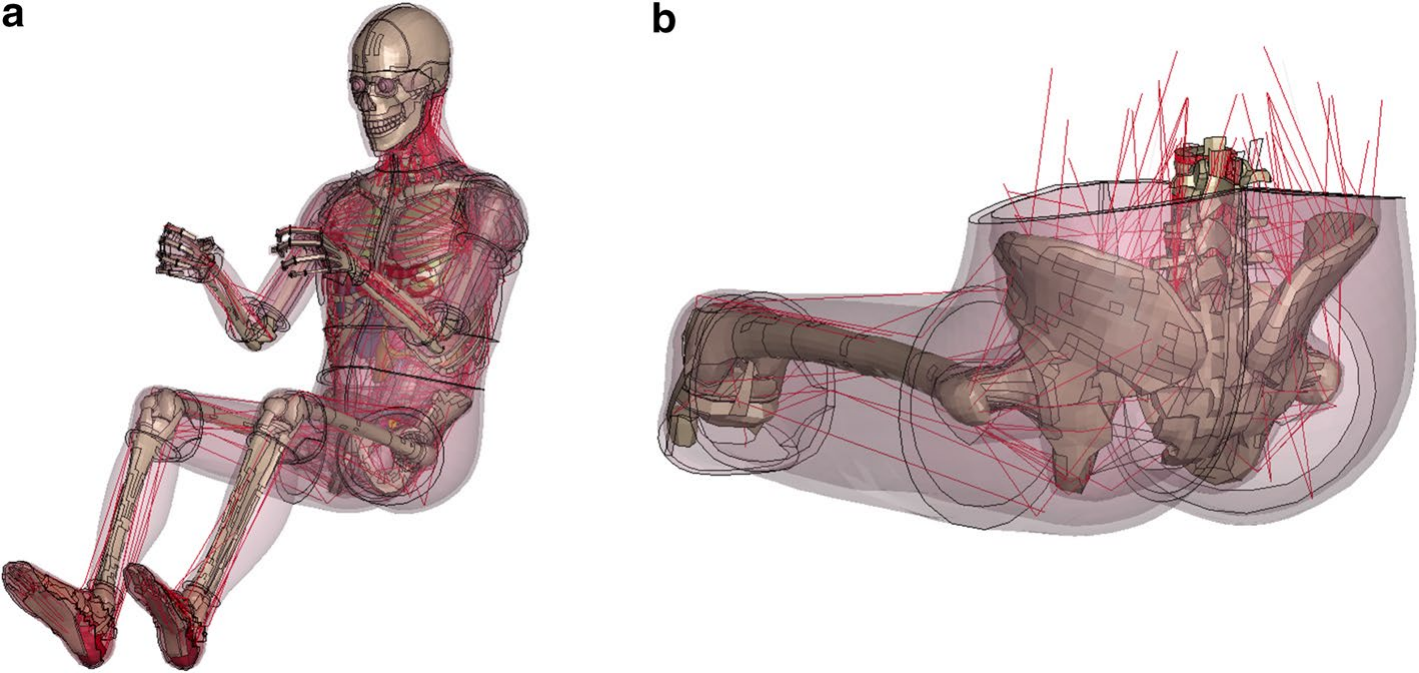
Colored depiction of THUMS Version 5.01 AM50 (**a**) and an example of the two muscle systems of THUMS (**b**): 1D system of Hill-type muscle truss elements (red lines) and the 3D system of volumetric, solid cuboid elements (transparent skin-colored parts)

In the past decades, several changes have been implemented to bring finite element models for the mechanical behavior of human bodies closer to biological reality. These human body models (HBM) now contain, e.g., facial bones, internal organs and a detailed spine model. The latest features are contractible, one-dimensional Hill-type muscle elements, allowing the HBM to perform musculoskeletal movements comparable to a living human being. Based on this implementation, the term ‘active human body model’ (AHBM) was established. Hill-type muscle elements follow an activation curve, which generates forces at the joints, thus enabling musculoskeletal movement and increasing the joint stiffness during simulation [2, 3]. The use of seatbelt elements to represent tendons and different combinations of sliprings allows the definition of complex paths, where each muscle is adjustable separately by individual activation curves [3]. This mechanism was implemented in the latest *Total HUman Model for Safety* (THUMS™) Version 5^1^ [2] and to a certain extent in other HBMs, e.g., for neck muscles in the open source VIVA model [4] or the models of the GHBMC family [5]. THUMS is one of the few models in which all major skeletal muscles (except facial muscles) were incorporated based on the 1D Hill-type muscle system (Fig. 1a) [6].

### Differences between muscle models and real muscles

Despite this complex modeling approach, certain features of biological muscles were hardly taken into consideration in the latest AHBM. One reason for this might be that biological muscles are too complex to implement every detail in the corresponding FE model, especially for multiple muscle systems; or that some of these might not be relevant for the analysis of mechanical muscle behavior [7] or AHBM applications. The features include anatomical, physiological, and material properties of muscles. Anatomical differences comprise the number and shape of the volumetric muscles. Approaches that address the issue in the context of HBM to a certain degree are the detailed GHBMC [8] and the Active THUMS [9]. Physiological differences include all biochemical and electrophysiological pathways and molecular interaction that provide the basis for muscle contraction and relaxation. To a certain extent, this issue was addressed in an ‘extended Hill-type muscle model’ for multiple Hill-type muscle models applicable for AHBM, considering Ca^2+^ levels [10] and a material model from the LS-DYNA library (MAT_ANISOTROPIC_HYPERELASTIC), considering, e.g., calcium concentration for the determination of active stresses of volumetric muscle elements [11]. Other models even considered single actin–myosin interactions on the molecular level [7, 12, 13], but due to their high complexity, these models are less suitable for multiple muscle [7] or HBM applications.

Differences regarding mechanical properties are the main topic of this work. Based on the modeling approaches in AHBM, we need to distinguish between two systems: On the one hand, a 1D system of Hill-type muscle truss elements, where passive and active muscle behavior from volunteer tests were implemented and optimized over the years [2, 6, 10]. The 1D system (Fig. 1b—red truss elements) can generate forces at the joints using predefined activation curves, thus enabling musculoskeletal movement and adjusting the joint stiffness during simulation. On the other hand, a 3D system of volumetric (solid) muscle elements, where the underlying material data were obtained from characterization experiments with Post Mortem Human Subjects (PMHS), as it is the case for the THUMS model [2, 14]. The 3D system depicts the volumetric shape of muscles, where multiple tissues were summarized mainly into two simplified parts (muscle and soft tissue) with a layered build-up, rather than representing an anatomically correct depiction of the shape of real individual muscles. For example, in case of the thigh muscles, two volumetric barrel-shaped parts surround the femur, representing muscle and soft tissues, which were modeled using identical material parameters (Fig. 1b, inner and outer layer of transparent skin-colored tissues). This layered build-up was used for nearly all volumetric muscle and soft tissues in THUMS. This work will only focus on the 3D system of volumetric muscle elements.

The data implemented in the corresponding LS-DYNA material models (e.g., MAT_SIMPLIFIED_FOAM) of volumetric muscle elements rather represent a cadaver than a living human, based on the validation data from PMHS tests. If a braced condition of an occupant is modeled, the stiffness of the 3D tissue (MAT_SIMPLIFIED_FOAM) should be higher than the cadaver tissue stiffness due to isometric contraction [15] to represent a living human realistically. The question is simply how much the stiffness should change between an entirely relaxed, a partly tensed and a tetanically contracted muscle. To answer this question, several studies on single muscles were conducted in the past and yet, no complete consensus was found. Different measurement techniques and muscle samples led to a vast amount of varying results, e.g., for the Young’s moduli of several orders of magnitude [16], as shown in Table 1 and Fig. 21. A consensus in the field of human muscle material characterization regarding measurement techniques, measurement parameters and muscle samples would be necessary to further exploit the capabilities of HBM. This would allow further development of multiple muscle models in the field of occupant safety by providing in vivo material data. The use of model populations to determine mechanical muscle properties for FE simulation can be a helpful tool in the process [17].

### Modeling approaches addressing volumetric muscle stiffness changes

Progress regarding contractible 3D muscles was recently made with the first forward dynamic contractible muscle system [18] of a biceps–triceps system. Other works rely on a coupled system, where contractible 1D truss elements are interwoven in 3D solid elements, resulting in a shape and stiffness change of the solid elements due to 1D contraction [9, 19–21]. This includes the development of the Active THUMS [9, 20]. Another study focused on the deformation processes under varying contraction states of a biceps [22]. These models take muscle stiffness changes into account based on different approaches. In the forward dynamic approach, a shape change resulting from the 3D contraction simulation leads to an increase in stiffness. The Active THUMS contains multiple 3D skeletal muscle reconstructed from MRI data that can change their shape due to contractible, interwoven 1D Hill-type muscle elements that share nodes with the 3D solid muscle elements. In consequence, this also leads to a change in muscle stiffness. This approach was applied to braced steering motion [20] and an entire HBM in pedestrian and occupant load cases [9, 21]. Another study connected a 3D muscle model of the lower extremities with the upper body of a Hybrid III dummy model for investigation of possible effects on occupant kinematics and acting forces [23]. However, these approaches do either not consider muscle stiffness on the material card level or might currently not be applicable to entire human body models in an efficient way with reasonable computation time.

The computation time is a crucial aspect for any finite element model and simulation, because it directly reflects the efficiency of the modeling approach. For any biomechanical model, a trade-off needs to be found between biological accuracy on the one hand and calculation speed and data evaluability on the other [7]. Some of these are assumed to be neglectable for mechanical problems in the field of impact biomechanics, such as physiological differences that were described earlier. The THUMS V5 was modeled in an efficient way to allow research and industrial applications. By doing so, certain simplifications were accepted by the developers [2], such as barrel-shaped volumetric muscle and soft tissues (Figs. 1b, 19) instead of anatomical muscle paths, muscle–tendon units and individually distributed fat tissues. Further, a hyperelastic material model (MAT_SIMPLIFIED_FOAM), described in detail in “Material model”, was used to model muscle and soft tissues of THUMS in a simplified way in LS-DYNA.

Other modeling approaches that showed certain advantages over the default THUMS material model were previously presented. On the one hand, the material model MAT_TISSUE_DISPERSED was used [24], which allows the simultaneous input of mechanical passive and active muscle tissue behavior, including the option to use activation curves as input to scale 3D muscle stiffness over time. With this approach, the authors could adequately model the muscle behavior obtained from experiment with a living rabbit [25]. On the other hand, MAT_ANISOTROPIC_HYPERELASTIC is one of the latest additions to the default LS-DYNA library, designed to represent biological soft tissues, specifically muscle tissues. The complexity of the material model can be easily altered according to the desired application and tissue type. Further, additional features, such as activation curve dependent stiffness scaling can be easily implemented [11]. The effect on the computation time of these modeling approaches, when implemented in human body models, is yet unknown.

### Aim of this work

The focus of this work addresses the question whether isometric contraction and the resulting change in muscle stiffness can be considered in a biofidelic way using the default material models of THUMS V5 (MAT_SIMPLIFIED_FOAM) without increasing the calculation time in view of efficient research and industrial applications of the altered THUMS model. As one of the most likely application fields of this AHBM adaption is the automotive sector, a simplified frontal impact simulation was chosen to analyze possible effects on occupant safety caused by muscle stiffness changes.

Based on a previously presented idea by the authors [1], the material stiffness of the 3D muscle tissue will be predefined. A material stiffness parameter is identified and altered for the thigh and the pelvis to define four different stiffness states. Further, to analyze the effect of a ‘worst case scenario’ on the computation time, the stiffness of all muscle and soft tissue of THUMS was modified for an exemplary simulation, not only for the pelvis and thigh. Additionally, a fifth state was defined, considering the highly strain-rate dependent passive properties of muscles (Table 1, Fig. 21), by incorporating data from Myers et al. [25] in all MAT_SIMPLIFIED_FOAM parts of THUMS by defining a Table ID to compare computation time and material properties.

The validatability of the simulation is limited by two factors. On the one hand, ethical reasons, as an experimental representation of the frontal impact simulations of this work including volunteers is not feasible and was not done to this extent in the past. On the other hand, literature data of PMHS tests cannot be used for validation due to physiological reasons, as isometric muscle contraction and the resulting, voluntary muscle stiffness change is no longer possible. Therefore, related literature [16, 26–29], where the kinematic and deformation behavior in loading experiments with volunteers was analyzed, was used for validation of the results of the frontal impact simulation. The effects of these stiffness changes on the tissue motion, peak impact force, computation time and the expected injury risk for the buttock, thigh and hip region will be analyzed in detail. As both, muscle and soft tissues, are modeled in a simplified way in THUMS (Fig. 19), both can be assumed to represent muscle tissue. Therefore, the stiffness of muscle and soft tissue of THUMS were scaled in the exact same manner.

The buttock, thigh and hip region (Fig. 19) were chosen for detailed investigation in this work, as they are relevant for lower extremity injuries occurring during frontal impacts due to the direct contact to the driver’s seat [30–33]. Most of the lower extremity injuries that result from automotive crashes occur in the knee, thigh and hip region [33–39]. According to data from 1993 to 1997 of the National Automotive Sampling System (NASS), 21.5% of all occupant injuries were accounted to lower extremity injuries [30]. Accordingly, they are the second most frequent injuries considered in the NASS database. Although lower extremity injuries are in most cases not life threating, they might result in long term hospitalization and physical disabilities [36–39]. Besides physical limitations, lower extremity injuries can have psychosocial long term effects, such as depression [38]. Both can cause high associated costs for the social sector. Further, this study was motivated by the hypothesis that one of the main function of muscles is the dissipation of mechanical energy after external impacts [40].

Based on these circumstances, further investigation in the field of lower extremity injuries and force effects on this body region during an automotive impact are necessary to allow the development of safer technologies and adaption of existing occupant safety regulations.

**Fig. 2:**
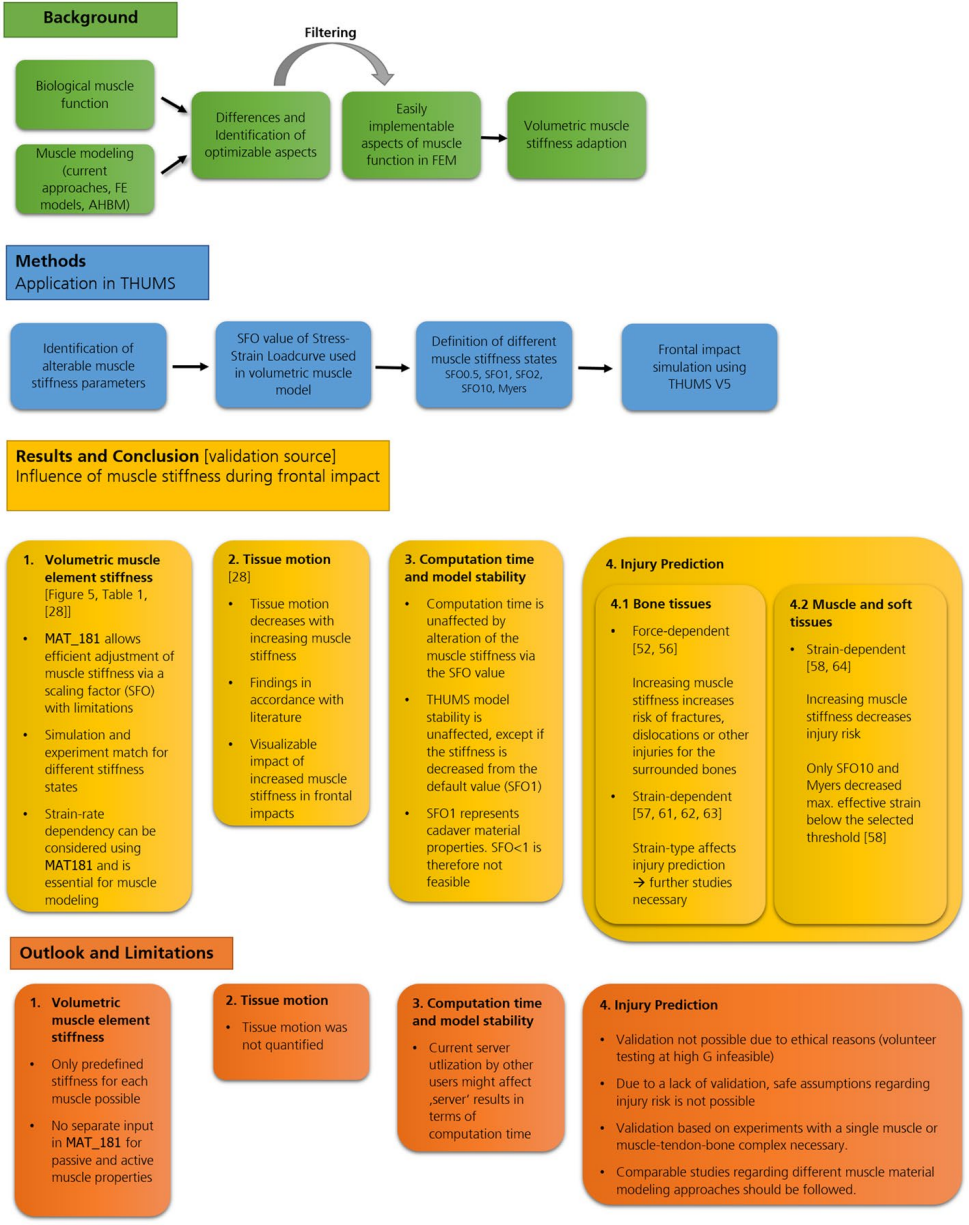
Overview of the respective sections and key findings of this study. MAT_SIMPLIFIED_FOAM (MAT_181) is the default material model used for modeling muscle and soft tissue in THUMS [3]

This work provides further insights into the possible effects of isometric contraction and the resulting muscle stiffness changes on injury outcome during frontal crash in the knee, thigh and hip region. Figure 2 shows an overview of the single chapters and key findings of this study.

## Employed simulation framework

The finite element method (FEM) is chosen for the numerical investigations of this work. The general method can be applied to find an approximate solution for an otherwise unsolvable structural mechanical problem, such as the frontal crash simulation of this work. In the following, a very brief introduction to the basic principles of the FEM is given and a material model, which is central to the described study, is presented. For more details on the subjects, the reader is referred to standard books in literature [41–45].

### Conservation equations and finite element method

For the following numerical investigations, the conservation of linear momentum is the essential differential equation that needs to be solved by numerical means. In its local form it is defined as:

1 $${\text{div }}{{\varvec{\upsigma}}} + { }\rho {\mathbf{k}} = \rho {\mathbf{a}},$$

with $\text{div}$ = divergence operator, ${\varvec{\upsigma}}$ = Cauchy stress tensor, $\mathbf{k}$ = spatial force vector, $\rho$ = density, and $\mathbf{a}$ = acceleration vector. This differential equation in its local form cannot be solved for every individual material point of a body and is therefore transferred into a weak formulation. Hence, Eq. (1) will only to be solved for the integral mean of a given domain, using the principle of virtual work (2). It states that the forces of inertia, resulting from the bodies’ motion and the internal forces must be in equilibrium with the external forces,

2 $$\begin{gathered} \delta W_{{{\text{kin}}}} + \delta W_{{\text{int }}} = { }\delta W_{{{\text{ext}}}} \hfill \\ \underbrace {{\mathop \smallint \limits_{{\text{V}}}^{{}} \rho {\mathbf{a}} \cdot \delta {\mathbf{u}}{\text{ d}}V}}_{{{\updelta }W_{{{\text{kin}}}} }} + \underbrace {{\mathop \smallint \limits_{V}^{{}} {{\varvec{\upsigma}}}:{\text{grad }}\delta {\mathbf{u}}{\text{ d}}V}}_{{{\updelta }W_{{\text{int }}} }} = \underbrace {{\mathop \smallint \limits_{{A_{t} }}^{{}} {\mathbf{t}}^{*} \cdot \delta {\mathbf{u}} {\text{d}}A + \mathop \smallint \limits_{{\text{V}}}^{{}} \rho {\mathbf{k}} \cdot \delta {\mathbf{u}}{\text{ d}}V}}_{{{\updelta }W_{{{\text{ext}}}} }}, \hfill \\ \end{gathered}$$

with $W$ = internal/external/kinetic energy, $\text{grad}$ = gradient operator, $\delta {\varvec{u}}$ = virtual displacements, ${\mathbf{t}}^{\mathbf{*}}$ = traction (stress acting on the surface of the body), $\text{d}V$ = differential of the volume, $\text{d}a$ = derivative of the acceleration,${\text{A}}_{t}$ = part of the surface area, where forces apply (Neumann boundary) and ‘$:$’ as the double dot product.

Using the finite element method, the domain of interest is now discretized into a number of elements with a finite element size. Each element is defined by a set of nodes with individual coordinates. From the displacements $\mathbf{u}$ of these nodes, the deformation of the parental element and in total, the deformation of the whole domain can be derived. Equation (2) can be transferred into a matrices equation, which can be formulated as a system of equations. By the application of shape functions ${\varvec{N}}$ required for the interpolation of the nodal coordinates and any given point within an element, Eq. (2) can be re-written to become numerically solvable:

3 $${\updelta }{\mathbf{u}}^{\top} \left[ {\underbrace {{\mathop \smallint \limits_{{\text{V}}}^{{}} {\varvec{N}}^{\top} \rho {\varvec{N}}{\text{ d}}V{{\ddot{\mathbf u}}}}}_{{\mathbf{M}}} + \underbrace {{\mathop \smallint \limits_{V}^{{}} {\varvec{B}}^{\top} {{\varvec{\upsigma}}}{\text{ d}}V}}_{{ {\mathbf{f}}_{{{\text{int}}}} }} - \left( {\underbrace {{\mathop \smallint \limits_{{A_{t} }}^{{}} {\varvec{N}}^{\top} {\mathbf{t}}^{*} {\text{d}}A + \mathop \smallint \limits_{{\text{V}}}^{{}} {\varvec{N}}^{\top} {\mathbf{k}} \rho {\text{ d}}V}}_{{ {\mathbf{f}}_{{{\text{ext}}}} }}} \right)} \right] = 0,$$

with ${{\varvec{B}}}^{\top }$ = transposed strain–displacement-matrix, $\ddot{\mathbf{u}}$ = second derivative of the nodal displacements (nodal acceleration vector) $\mathbf{M}$ = mass matrix,${\mathbf{f}}_{\text{int}}$ = internal forces and ${\mathbf{f}}_{\text{ext}}$ = external forces. (3) can be written in its simplified form:

4 $${\updelta }{\mathbf{u}}^{\top} \left[ { {\mathbf{M}} {{\ddot{\mathbf u}}} + {\mathbf{f}}_{{{\text{int}}}} { } - {\mathbf{f}}_{{{\text{ext}}}} } \right] = { }0.$$

(4) is solved for each element in discrete time steps using the explicit time integration method. Hereby, data for the future time step $(t+\Delta t)$ are calculated from the data available at the current time step $(t)$. Since no convergence criterion has to be fulfilled for explicit time integration, the only limit is being set by the maximum critical time step assuring the conditional stability of the scheme, by using the Courant–Friedrichs–Lewy (CFL) criterion

5 $$\omega_{{{\text{max}}}} = { }\frac{2c}{L} \mathop \Rightarrow \limits_{{}} \Delta t < \Delta t_{{{\text{krit}}}} = \frac{L}{c} = \frac{2}{{\omega_{{{\text{max}}}} }},$$

where *ω*_max_ = critical eigenfrequency, c = speed of sound, *L* = element’s length and Δ*t*_krit_ = maximum value of the time step, respectively [41, 43, 46].

### Material model

Material properties of the soft and muscle tissue that are modeled using the material model MAT_SIMPLIFIED_RUBBER/FOAM were employed and strongly varied in this study. The material model can behave rubber-like or foam-like, depending on the value of the Poisson ratio $v$ [47].

If a Poisson’s ratio $v$ between 0 and 0.5 is defined, which is the case in THUMS, then the model acts as a hyperelastic, isotropic, compressible foam-like material (MAT_SIMPLIFIED_FOAM). The Hill functional ${\Psi }_{\text{Hill}}$ is used and the Poisson’s ratio ($v$) determines the penalty term of pressure in the functional. Further, the bulk modulus $K$ is only used to determine the time step, for contact definitions and for hourglass stiffness.

The Hill functional—not to be confused with the Hill-type 1D muscle model addressed in “Background”—is defined in dependency of the stretch tensor $\mathbf{C}$ or the principle stretches ${\lambda }_{1}, {\lambda }_{2}$ and ${\lambda }_{3}$:

6 $$\begin{gathered} \Psi = \Psi \left( {\mathbf{C}} \right) = \Psi_{{{\text{Hill}}}} \left( {\lambda_{1} , \lambda_{2} ,\lambda_{3} } \right), \hfill \\ \Psi_{{{\text{Hill}}}} = \mathop \sum \limits_{j = 1}^{m} \frac{{C_{j} }}{{b_{j} }}\left[ {\lambda_{1}^{{b_{j} }} + \lambda_{2}^{{b_{j} }} + \lambda_{3}^{{b_{j} }} - 3 + \frac{1}{n}\left( {J^{{ - nb_{j} }} - 1} \right)} \right] , \hfill \\ \end{gathered}$$

with ${C}_{j}$, ${b}_{j}$ and $n$ as material constants and the Jacobian determinant $J= {\uplambda }_{1} {\uplambda }_{2}{\uplambda }_{3}$ according to [47, 48] describing the volumetric change. The parameter $n$ is directly dependent on the Poisson’s ratio $v$ and can be calculated by the relation

7 $$n = { }\frac{v}{2v - 1}.$$

In the functional, the term $\frac{1}{n}\left({J}^{{-nb}_{j}}-1\right)$ defines the penalty term of pressure. From Eq. (6), the principal Cauchy stress components ${\sigma }_{a}$ can be derived:

8 $$\sigma_{a} = J^{ - 1} \lambda_{a} \frac{\partial \Psi }{{\partial \lambda_{a} }} \forall a \in \left\{ {1,2,3} \right\}.$$

The eigenvectors ${\mathbf{n}}_{a}$ result from the spectral decomposition of the stretch tensor $\mathbf{C}$. By calculation of the outer product, the second order stress tensor ${\varvec{\upsigma}}$ can be calculated from the eigenvectors ${\mathbf{n}}_{a}$ and from the principle Cauchy stresses ${\sigma }_{a}$:

9 $${{\varvec{\upsigma}}} = { }\mathop \sum \limits_{a = 1}^{3} \sigma_{a} {\mathbf{n}}_{{\varvec{a}}} \user2{ } \otimes \user2{ }{\mathbf{n}}_{{\varvec{a}}} = J^{ - 1} \lambda_{a} \frac{\partial \Psi }{{\partial \lambda_{a} }} {\mathbf{n}}_{{\varvec{a}}} \user2{ } \otimes \user2{ }{\mathbf{n}}_{{\varvec{a}}} .$$

Force–displacement data obtained from experiments can be used as input for the material model. If the gauge length, width and thickness are calibrated (equal to 1), engineering stress and strain curves are used as input or tables, which reference different curves for e.g., different strain rates. This material model was used and altered for variations in muscular stiffness in the buttock, thigh and hip joint muscle and soft tissue elements of THUMS [41, 49–51].

## Results

**Fig. 3:**
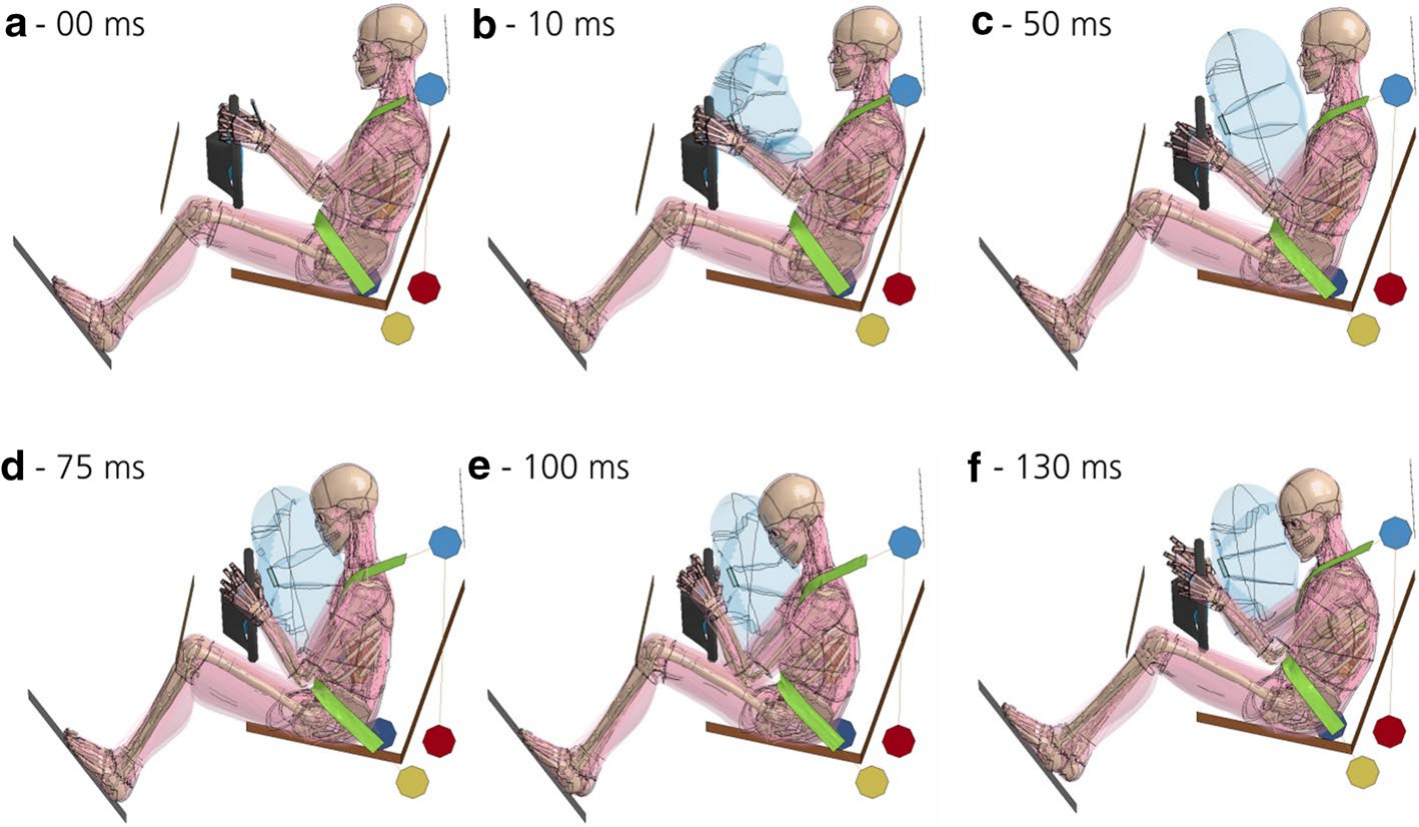
Simplified frontal crash pulse with THUMS. A simplified vehicle model is used. For this graph, the default muscle stiffness of THUMS was used for the muscle and soft tissues

A general overview of the frontal impact simulation is shown in Fig. 3. The events occurring over time are described in detail in Appendix.

In the following, the effect of different volumetric muscle tissue stiffnesses is shown in comparison with literature regarding resulting stress–strain curves during impact, tissue motion, computation time and injury prediction. To achieve different muscle tissue stiffnesses, the ordinate value (stress value) of the engineering stress–strain curve was scaled, which is defined within the hyperelastic material model MAT_SIMPLIFIED_RUBBER/FOAM used to model muscle and soft tissues in THUMS. Different values of this scaling factor of the ordinate value (SFO) were selected to achieve different stiffness states, as further described in “Methods” of this work.

Each of the following subsections will be discussed in the respective subsection in “Discussion”.

### Volumetric muscle element stiffness

**Fig. 4:**
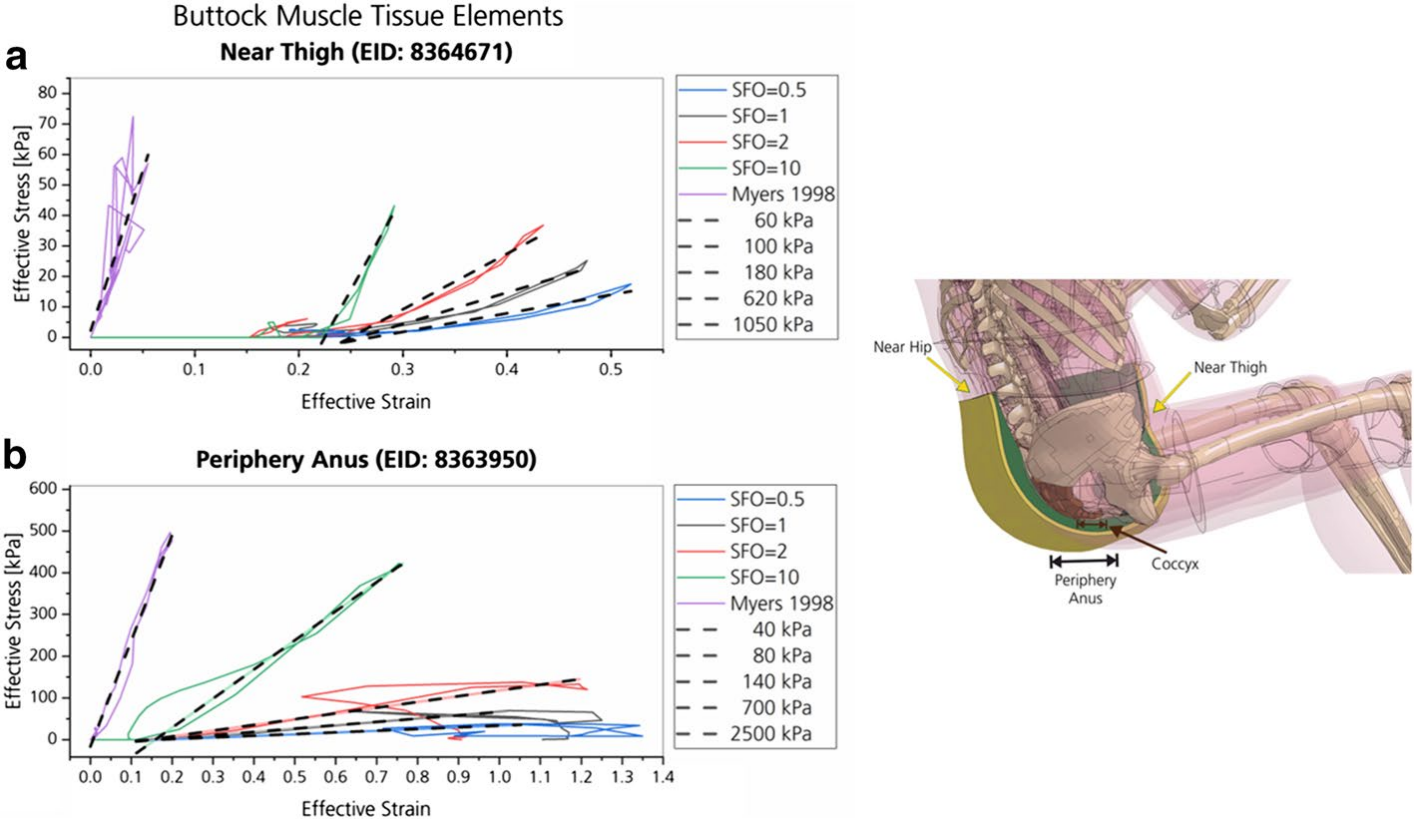
Effective stress–strain curves of the outer muscle tissue of the buttock including linear fits (dashed lines) for approximation of the Young’s moduli (kPa) for each scaling factor. Modeled with MAT_SIMPLIFIED_FOAM

As shown in Fig. 4, the slope of the effective stress–strain curve for elements of the buttock and thigh region increases with increasing SFO values. The element stiffness is directly defined by the slope of this curve. The slope of the linear fits, calculated for the timespan of highest loading during impact, approximates the Young’s modulus for each curve. The slope increases with the SFO value, but not proportionally. Arrows indicate points of maximum effective stress and strain. Literature data [25] incorporated in the model showed the highest slope in comparison [25].

**Fig. 5:**
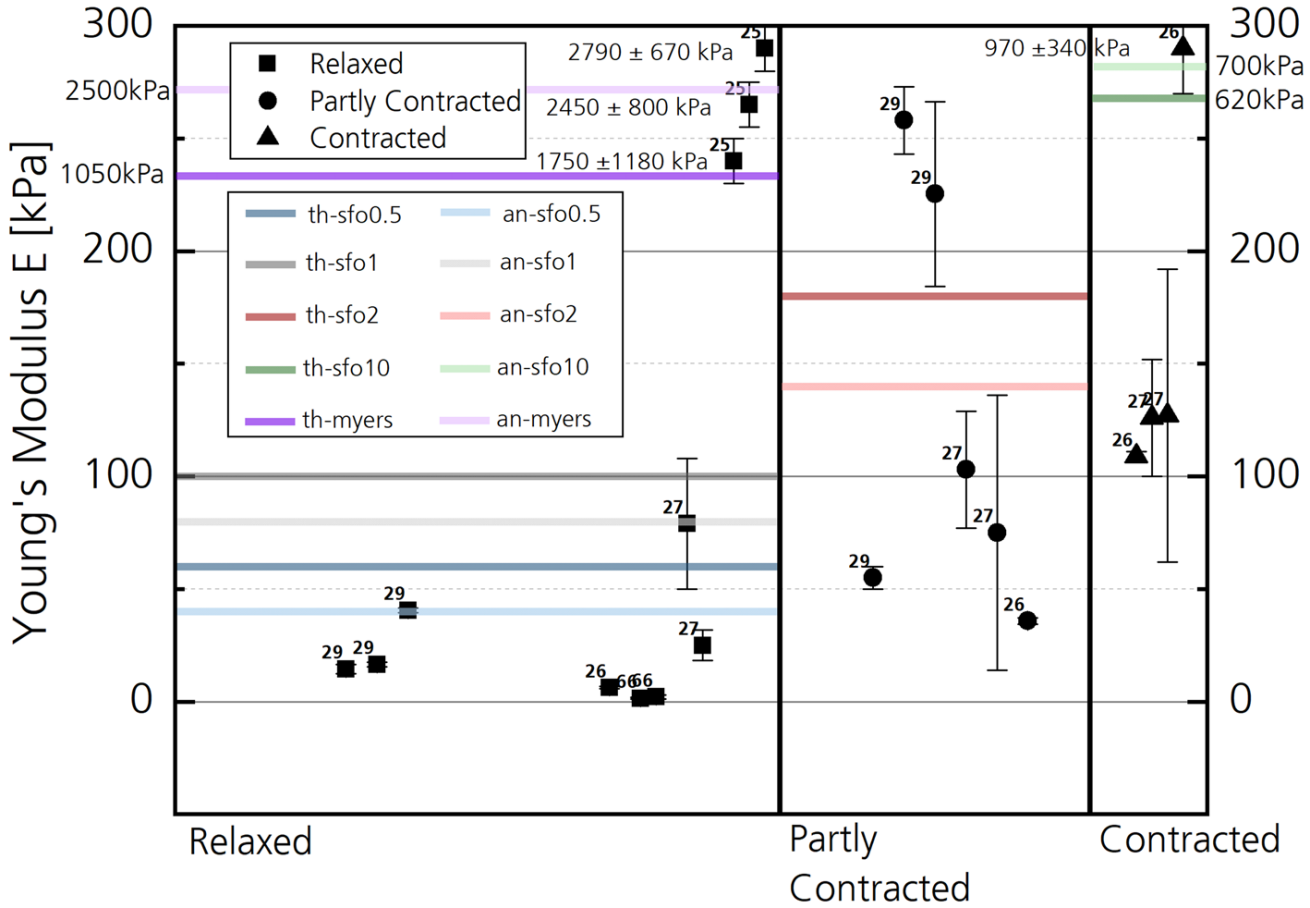
Comparison of Young’s moduli for muscles from literature (dots) and numerical simulation (lines) of this work. For the numerical data, the Young’s moduli were calculated from linear fits of the stress–strain curves of the respective elements shown in Fig. 4. Data from ‘*near thigh*’ are abbreviated with ‘*th*’, ‘*periphery anus*’ with ‘*an*’*.* Literature data include different contractile states of different muscles (in vivo) or muscle samples (ex vivo) from humans, bovines and porcines. Indicators refer to the respective literature source, referenced and further explained in Table 1. Additional literature data on Shear moduli are shown in Fig. 21. The majority of the literature data shown were summarized prior to this study [40]. Apart from the subdivision in different contractile states, there is no correlation on the abscissa of the data points. Distances of data points within one group are arbitrary

In Fig. 5, Young’s moduli of different human and animal muscles from literature data are compared to those calculated from numerical simulation (Fig. 4) for different material stiffness parameters. The data are subdivided into three regions of entirely independent data. From left to right, data on ‘relaxed’, ‘partly contracted’ and ‘contracted’ muscle samples are shown. ‘Relaxed’ includes experimental data from ex vivo experiments from, e.g., isolated bovine and porcine muscle, as well as in vivo human muscle measurements without voluntary contraction. ‘Partly contracted’ includes muscle stiffness states at various levels (e.g., 20%, 30% voluntary contraction, lifting of 7.5 kg weight, etc.) from human volunteers. Data classified as ‘contracted’ includes experimental data from human volunteers, during which the muscle was contracted to the maximum voluntary level or where heavy weights were lifted (15 kg). Further details can be found in Table 1 of Appendix. Experiments during which tetanic excitation was reached in rabbit *tibialis anterior* muscle by nerve excitation was also assigned to the ‘contracted’ samples [25]. An averaged Young’s modulus of different strain rates in the ‘contracted’ state is shown, as well as separate values of Young’s moduli for different strain rates at the ‘relaxed’, passive muscle state.

Results from SFO0.5 and SFO1 were both classified as ‘Relaxed’. SFO2 as ‘Partly contracted’ and SFO10 as ‘Contracted’. As the Young’s modulus of SFO10 was much higher than in the literature data, respective values are listed separately. The same accounts for literature values of [25], who performed their experiments at higher strain rates than the other sources, resulting in much higher Young’s moduli. Data to the presented injury risk curve [25] were incorporated in MAT_SIMPLIFIED_FOAM via a Table ID and are shown as the stress–strain curve ‘Myers 1998’, cf. Fig. 4. Linear fits, approximating the Young’s modulus, are in range of the literature data (Fig. 5) obtained during tensile tests [25].

### Tissue motion

**Fig. 6:**
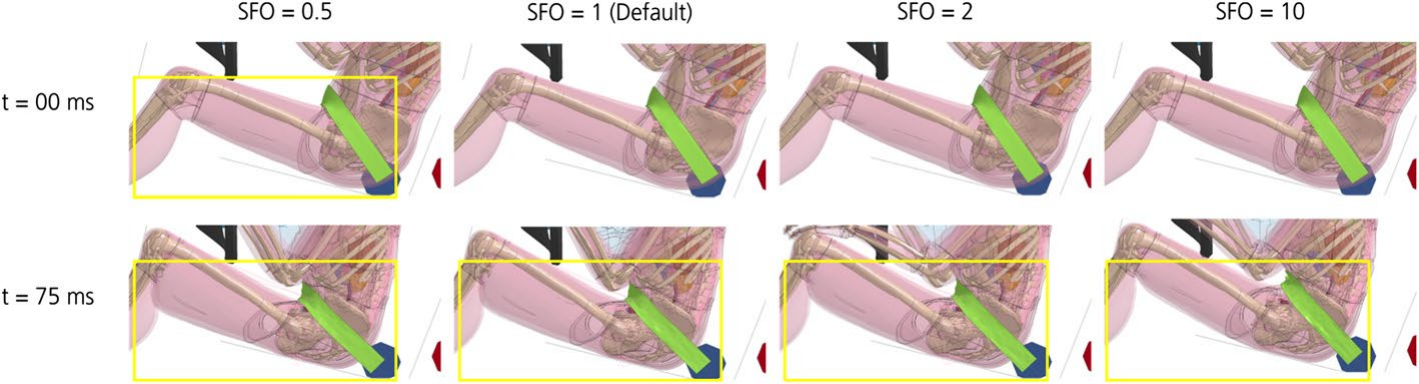
Effect of a change in SFO on tissue motion. The effect on the muscle and soft tissue motion of the buttock and thigh after 75 ms is shown (yellow-framed). Decreasing tissue motion with increasing muscle stiffness parameter value can be observed. Differences in the arm position probably occurred due to numerical variations as the result of the change in SFO, which led to an early slipping of the hands off the steering wheel for some SFO cases. For SFO2 and SFO10, both hands slip off. For SFO0.5, only the right hand slips off

**Fig. 7:**
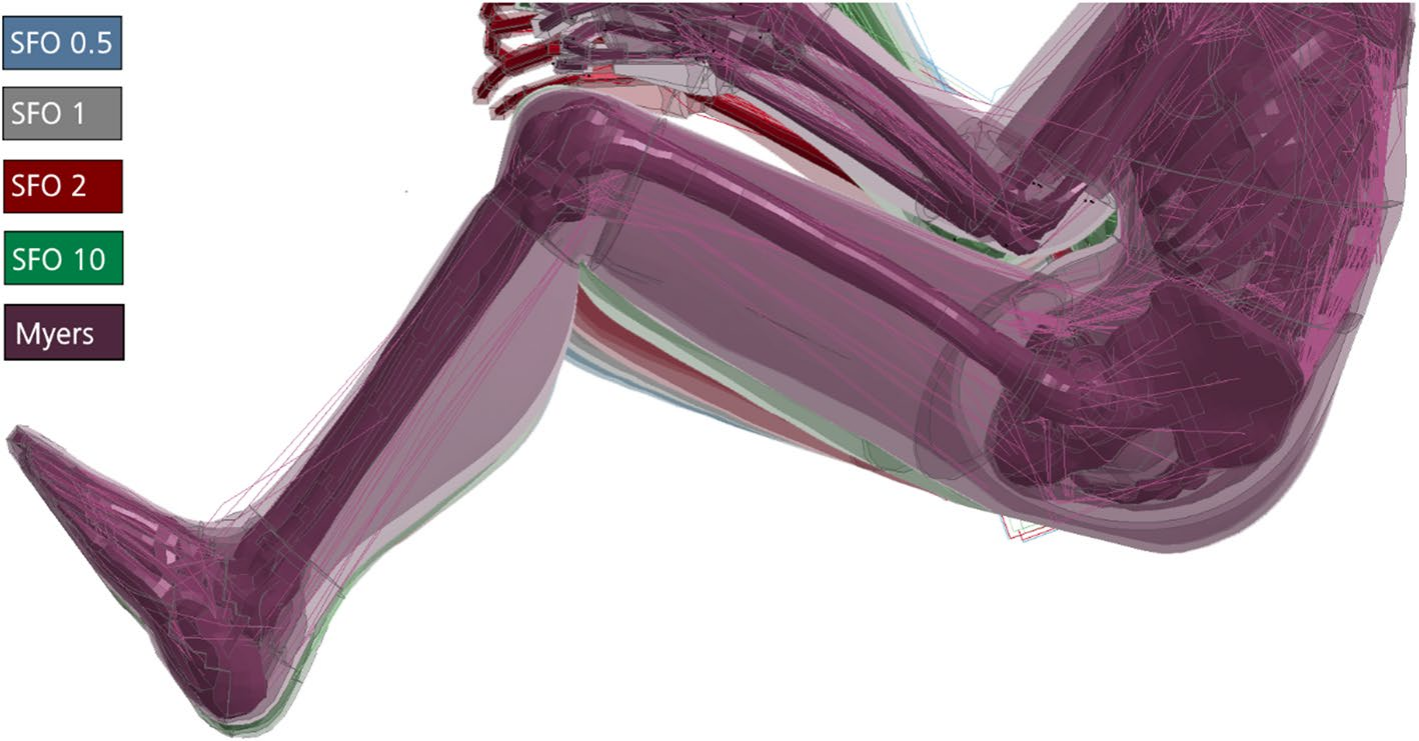
Comparison of tissue motion at different muscle and soft tissue stiffnesses. Tissue motion is decreasing with increasing muscle and soft tissue stiffness. For ‘Myers’, passive strain-rate dependent data [25] were incorporated into all volumetric muscle and soft tissues of THUMS, not only the ones in the pelvis and thigh

If the material stiffness parameter (SFO value) is increased, the tissue motion caused by the frontal impact is reduced. This can be observed in Fig. 6 in the distal region of the thigh, close to the knee joint. Only optical evaluation on tissue motion based on Figs. 6 and 7 was performed.

### Computation time and model stability

**Fig. 8:**
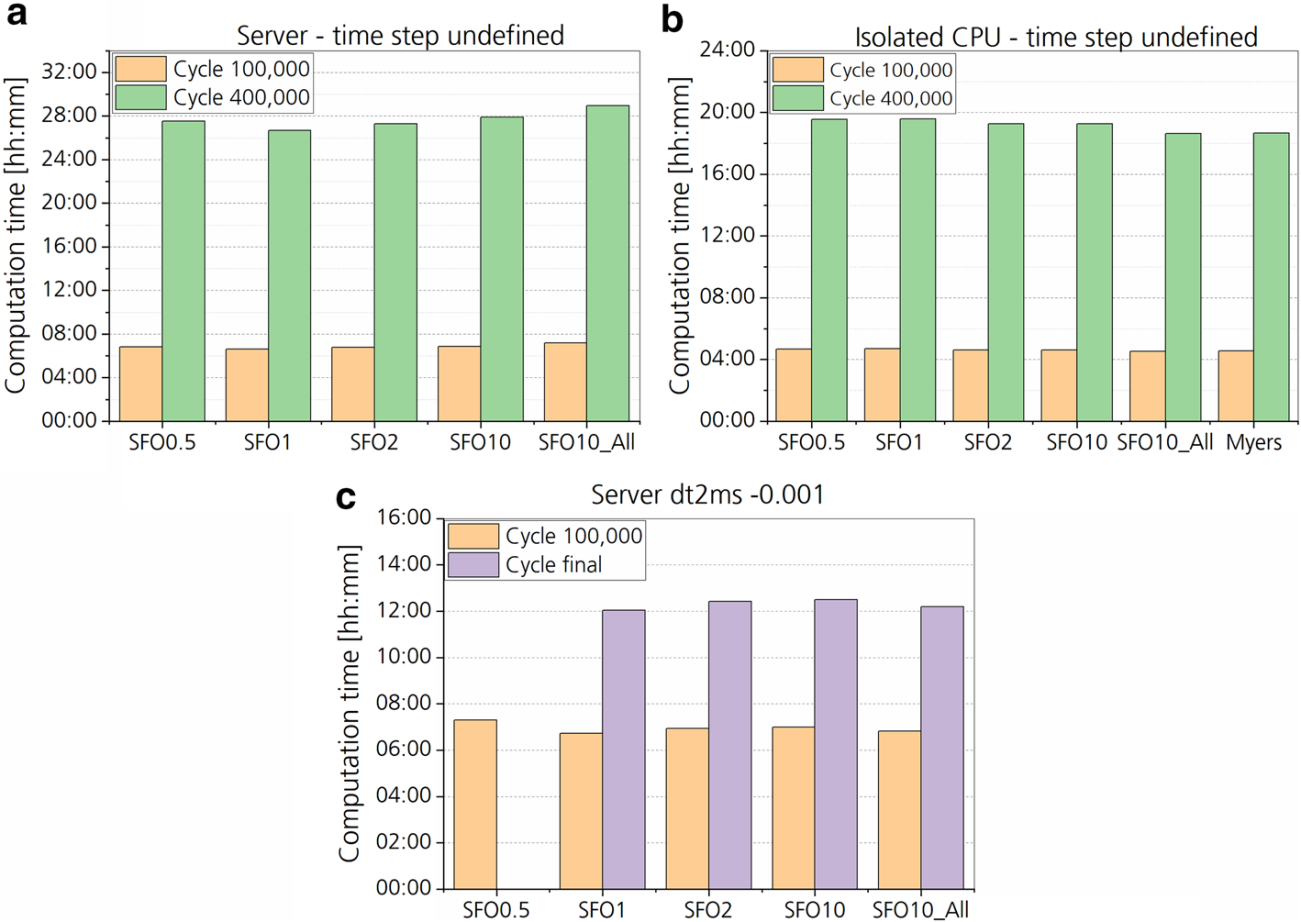
Computation time of the frontal impact simulation for different muscle stiffness states and for different keyword and hardware settings. For ‘Server’, Input and Output (IO) and CPU performance were influenced by simulations of other users, while for ‘Isolated CPU’ only IO was influenced. Data from all different stiffness cases are shown. Cycle refers to one cycle of the explicit time integration. **a** and **b** did not reach the final simulation time of 160 ms. Therefore, the computation time was compared for specific cycles. **a** Simulation was run on a server with undefined time step in the CONTROL_TIMESTEP keyword. **b** Simulation was run on an isolated CPU with undefined time step. **c** Simulation was run on a server with a predefined time-step size of 1 ms achieved by mass scaling

**Fig. 9:**
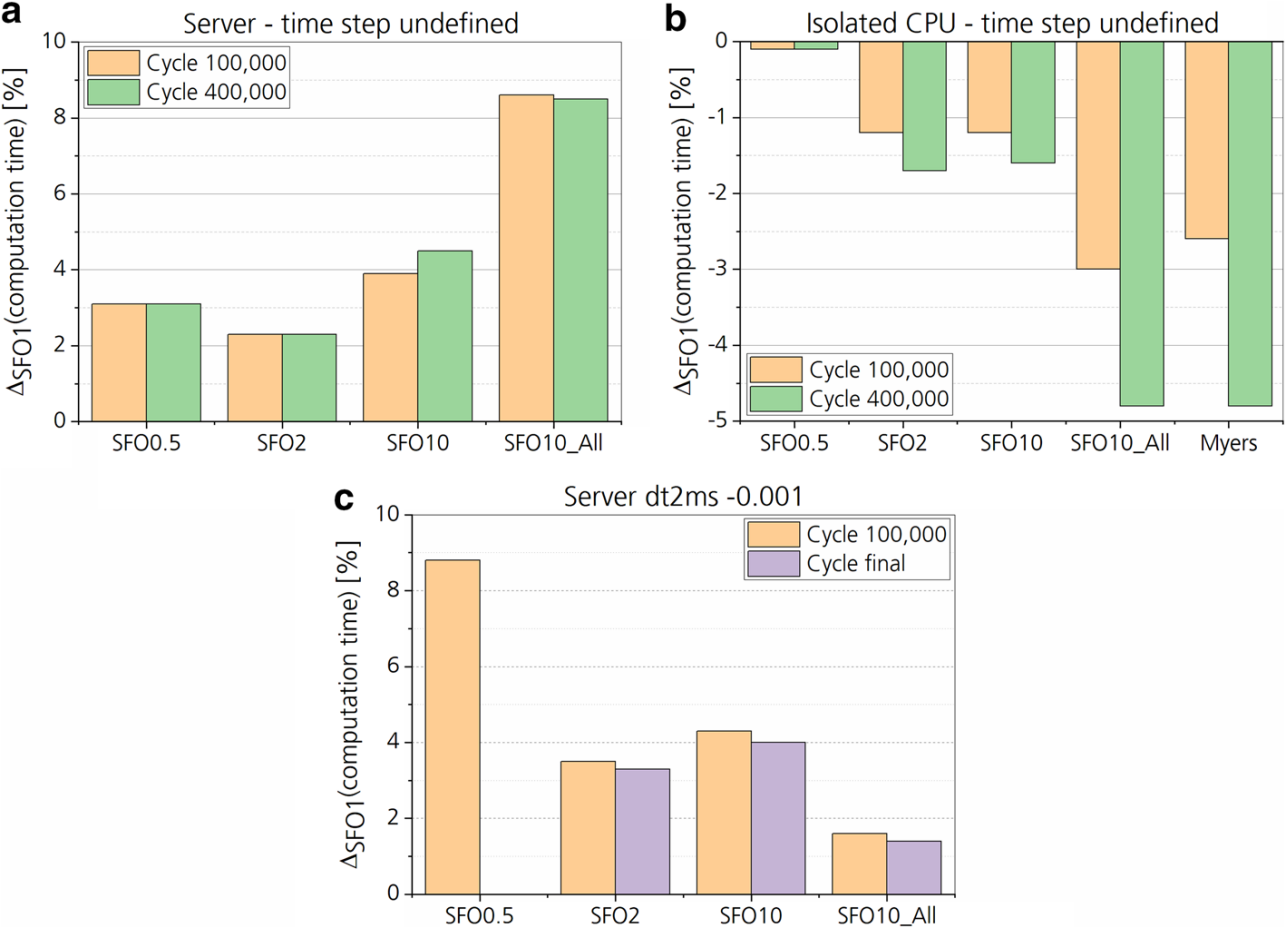
Computation time in percent for all stiffness states compared to the default stiffness state (SFO1). The same comparison regarding keyword and hardware settings and stiffness cases as in Fig. 8 is shown. Error terminations occurred for all simulations with undefined time step before the final termination of 160 ms. Therefore, the final cycles could not be compared regarding computation time, but only the cycle 100,000 (~ 20 ms) and cycle 400,000 (~ 55 ms). Error termination also occurred for the SFO0.5 case (dt2ms − 0.001) after 125 ms due to negative volume error in the transition area between knee and tibia. **a** Simulation was run on a server with undefined time step in the CONTROL_TIMESTEP keyword. **b** Simulation was run on an isolated CPU with undefined time step. **c** Simulation was run on a server with a predefined time-step size of 1 ms achieved by mass scaling

Varying the stress–strain load curve (LC) from the default value (SFO = 1) hardly had an impact on the computation time (Figs. 8, 9). The largest difference with approximately 8% to the default SFO1 case was found for the SFO0.5 case with a predefined time-step size of 0.001 ms achieved by selective mass scaling. Generally, the current server utilization by other users and processes as well as hyperthreading seemed to have a much higher impact on the computation time than the change in material model parameters. This was found for the four different SFO values and ‘Myers’, which were calculated multiple times at various states of server utilization. Depending on the state of server utilization, computation times varied by several hours. Differences in computation time were smallest for the isolated CPU. Some of the simulations with undefined time-step size were error-terminated after a minimum computation time of 60 ms (SFO0.5, Isolated CPU). All computations passed cycle 100,000 and 400,000, which were therefore used for computation time comparison.

Results from the simplified cuboid model showed that any changes in SFO value or stress–strain curve including different strain rates (Myers) had no significant impact on the computation time. SFO0.5 again showed the highest decrease in computation time of 3.1% compared to SFO1, while computation time of SFO2 decreased by 0.2% and of SFO10 by − 1.1%. Myers had the highest increase of 2.0%. The final number of cycles calculated was constant for all material parameter variations, for both defined and undefined timestep size. For all cuboid model simulations normal termination occurred.

Regarding model stability, all simulations reached the final calculation cycle of explicit time integration (160 ms simulation time) for the mass-scaled solution (dt2ms), except the SFO0.5 simulation, which was error-terminated after 125 ms due to negative volume errors in the knee region. Therefore, reducing the muscle stiffness resulted in model instabilities.

### Injury prediction

Results regarding injury prediction will be presented in two subsections: injuries of bone tissues and injuries of muscle and soft tissues. Resultant forces (contact forces), first principal strain and effective plastic strains were used as injury criteria for injury prediction.

#### Bone tissues

##### Force-dependent injury prediction

The seat bottom was exclusively in contact with the THUMS pelvis and proximal region of the thigh. The knee bolster was exclusively in contact with the knee. Resultant forces were obtained from contact forces as described in subsection “Injury prediction”.

**Fig. 10:**
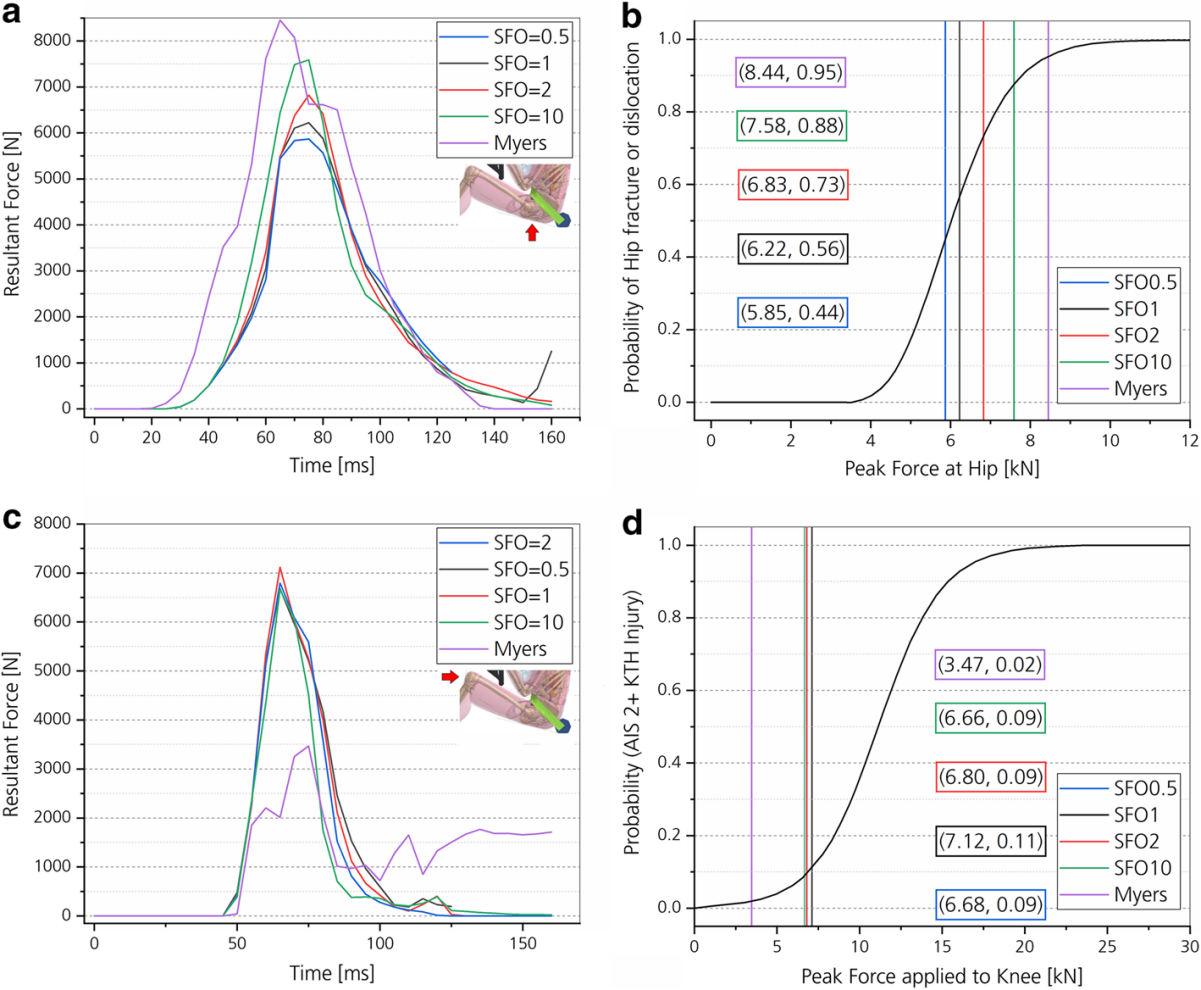
Resultant forces and injury probabilities for the hip and knee region dependent on muscle stiffness. Peak impact force between the THUMS skin and the seat bottom (**a**) and the THUMS skin and knee bolster (**c**) are shown over time. For ‘Myers’, passive strain-rate-dependent data [25] were incorporated into all volumetric muscle and soft tissues of THUMS, which includes the volumetric tissue surrounding the knee in contrast to the other stiffness simulations. Injury risk for the hip (**b**) and the knee (**d**) during frontal impact for different muscle stiffnesses are shown. Maximum contact force values from images **a** and **c** are plotted. The injury risk curve was developed by Rupp et al. [52] based on frontal impact testing of isolated pelvis and lower extremity complexes

Contact forces between the THUMS skin and the vehicle parts and the resulting injury probability are shown in Fig. 10. For the buttock, the peak resultant force increases with increasing SFO value, but not by the same factor. The highest peak force between buttock and seat was found after 75 ms for all stiffness values except the Myers stiffness value. For the latter, the peak resultant force is reached after 65 ms. Increasing muscle stiffness accordingly leads to an increasing probability of hip fracture or dislocation according to the presented injury risk curve [52].

The muscle and soft tissue stiffness changes in the hip and thigh region do not have an impact on the probability of knee injuries (Fig. 10c, d, all except purple). For SFO0.5, SFO1, SFO2 and SFO10 the stiffness of knee surrounding muscle and soft tissues were unchanged from the default settings. For the Myers stiffness case, higher tissue stiffness was defined for all volumetric muscle and soft tissue elements modeled with MAT_SIMPLIFIED_FOAM, including the tissues surrounding the knee joint. Increasing the stiffness of knee surrounding tissues reduced the probability of knee injuries (AIS2+) from 9–11 to 2%.

##### Strain-dependent injury prediction

The threshold of 3% ultimate strain was hardly exceeded by any cortical or spongy bone elements when analyzed regarding effective plastic strains and first principal strains. Therefore, a lower limit of 1.5% was selected to allow a better comparison of muscle stiffness effects on the predicted injury risk of the bones. The volume of shell and solid elements that failed the threshold of 1.5% effective plastic strain or first principal strain was added up, resulting in a ‘failed element volume’. Depending on the strain type and bone tissue, different impact of muscle stiffness on injury risk prediction can be found.

**Fig. 11:**
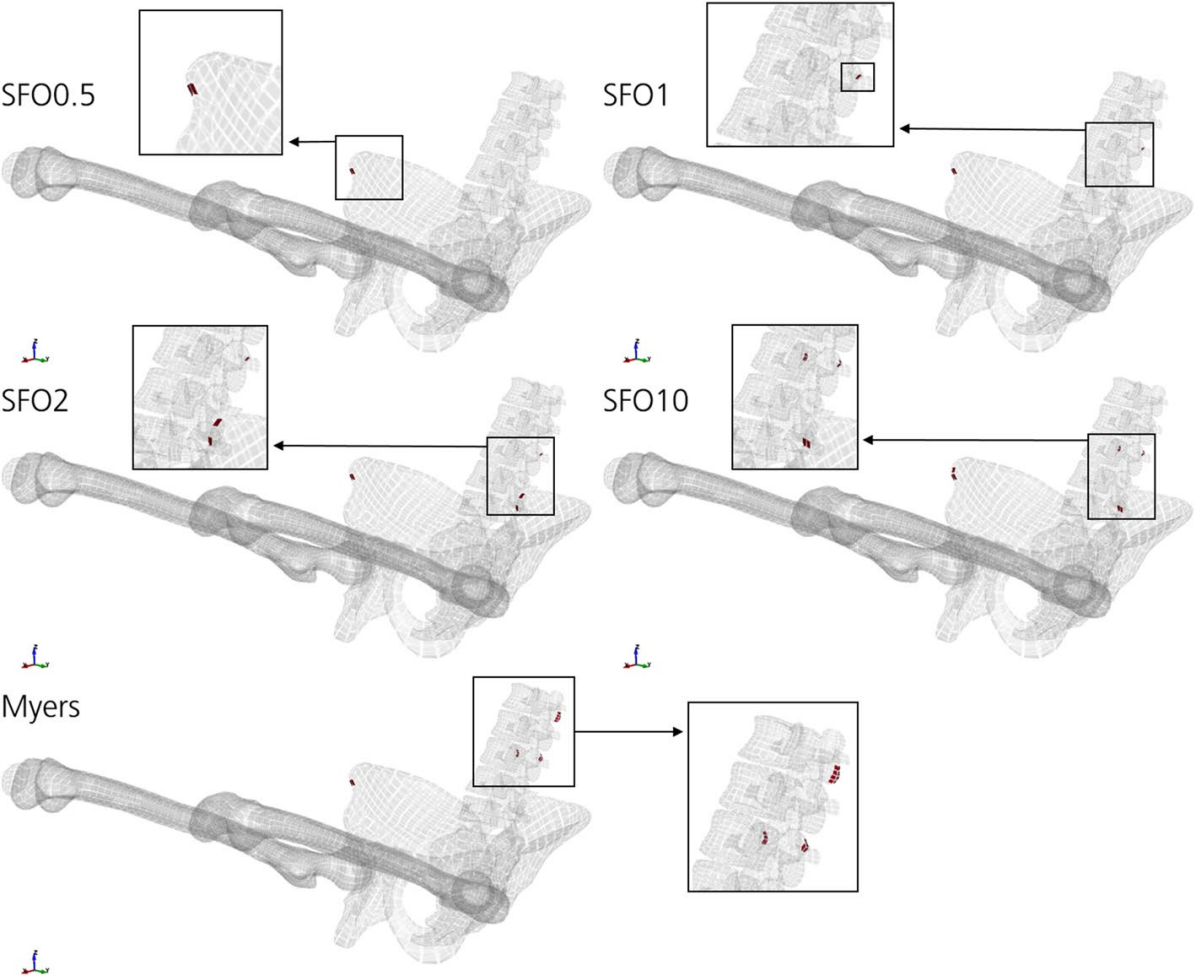
Cortical bone elements with effective plastic strain greater than 1.5% accumulated over the entire computation time. The threshold was exceeded in the following body regions: right hipbone 1.5 mm thickness (all simulations), 3rd lumbar vertebrae posterior (all except SFO0.5), left hipbone 1.5 mm thickness (SFO2, SFO10), 1st lumbar vertebrae posterior (Myers). The volume of failed shell elements accumulated to 87 mm^3^ (SFO0.5), 103 mm^3^ (SFO1), 223 mm^3^ (SFO2), 275 mm^3^ (SFO10) and 247 mm^3^ (Myers)

Data on effective plastic strains of cortical bones (Fig. 11) show that the predicted injury risk based on failed element volume is increasing with increasing muscle stiffness, as well as the number of locations, where the threshold of 1.5% strain is exceeded. In contrast, the Myers stiffness case which is stiffer than SFO10 (Fig. 4) has a slightly lower injury risk based on failed element volume (Fig. 11). Body regions that exceeded the threshold were the right hipbone 1.5 mm thickness (all simulations), 3rd lumbar vertebrae posterior (all except SFO0.5), left hipbone 1.5 mm thickness (SFO2, SFO10) and the 1st lumbar vertebrae posterior (Myers).

**Fig. 12:**
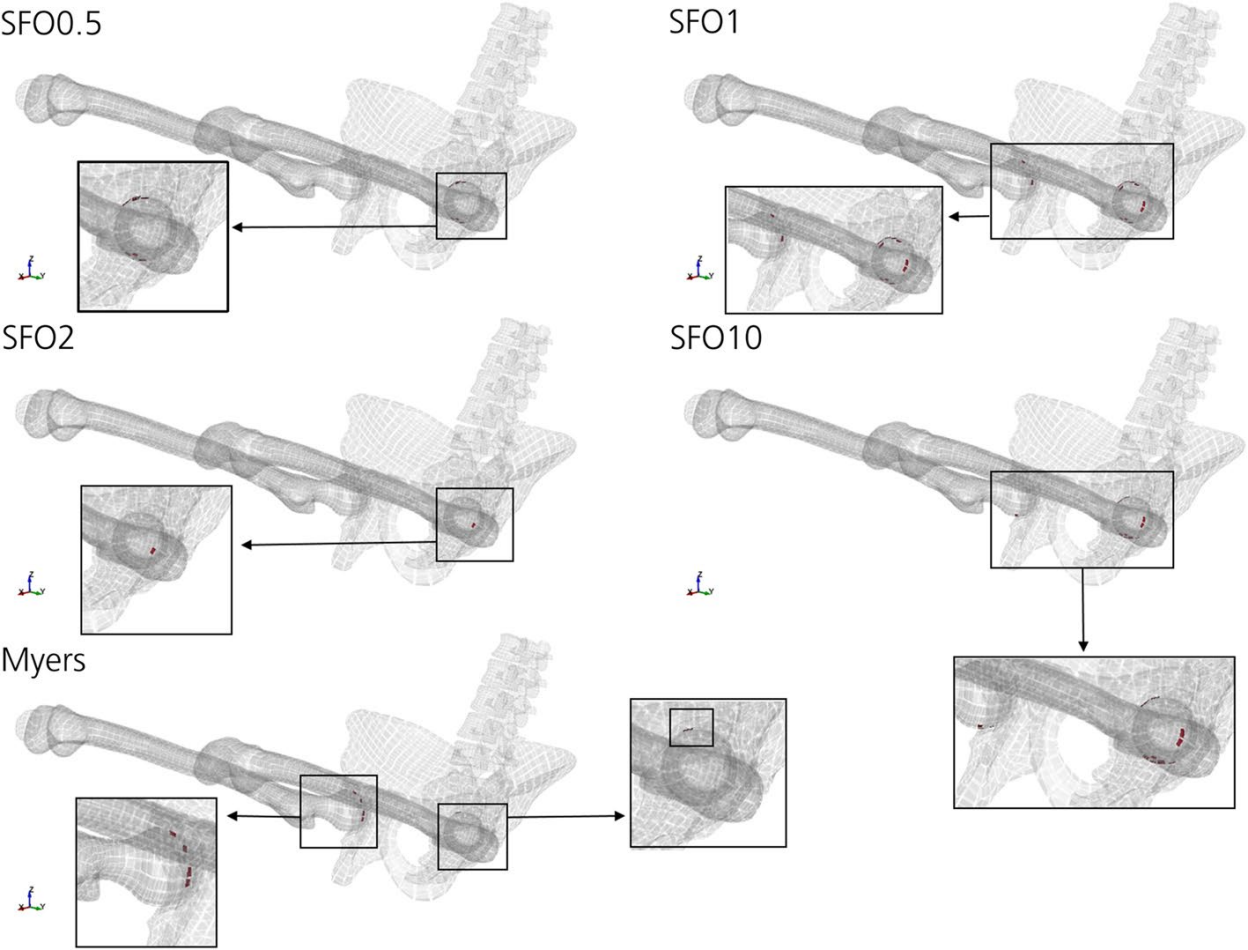
Cortical bone elements with 1st principal strain greater than 1.5% over the entire computation time. The threshold was exceeded in the following body regions: left femur upper null shell (all except Myers), right femur upper null shell (SFO1, Myers). Peak volumes of failed elements were 134 mm^3^ (SFO0.5, 110 ms), 140 mm^3^ (SFO1, 120 ms), 28 mm^3^ (SFO2, 150 ms), 206 mm^3^ (SFO10, 70 ms), 66 mm^3^ (Myers, 75 ms)

Data on first principal strains of cortical bones (Fig. 12) show an increase in predicted injury risk based on failed element volume in the following order: SFO2, Myers, SFO0.5, SFO1 and SFO10. Affected body regions of THUMS were the left femur upper null shell (all except Myers) and the right femur upper null shell (SFO1, Myers).

**Fig. 13:**
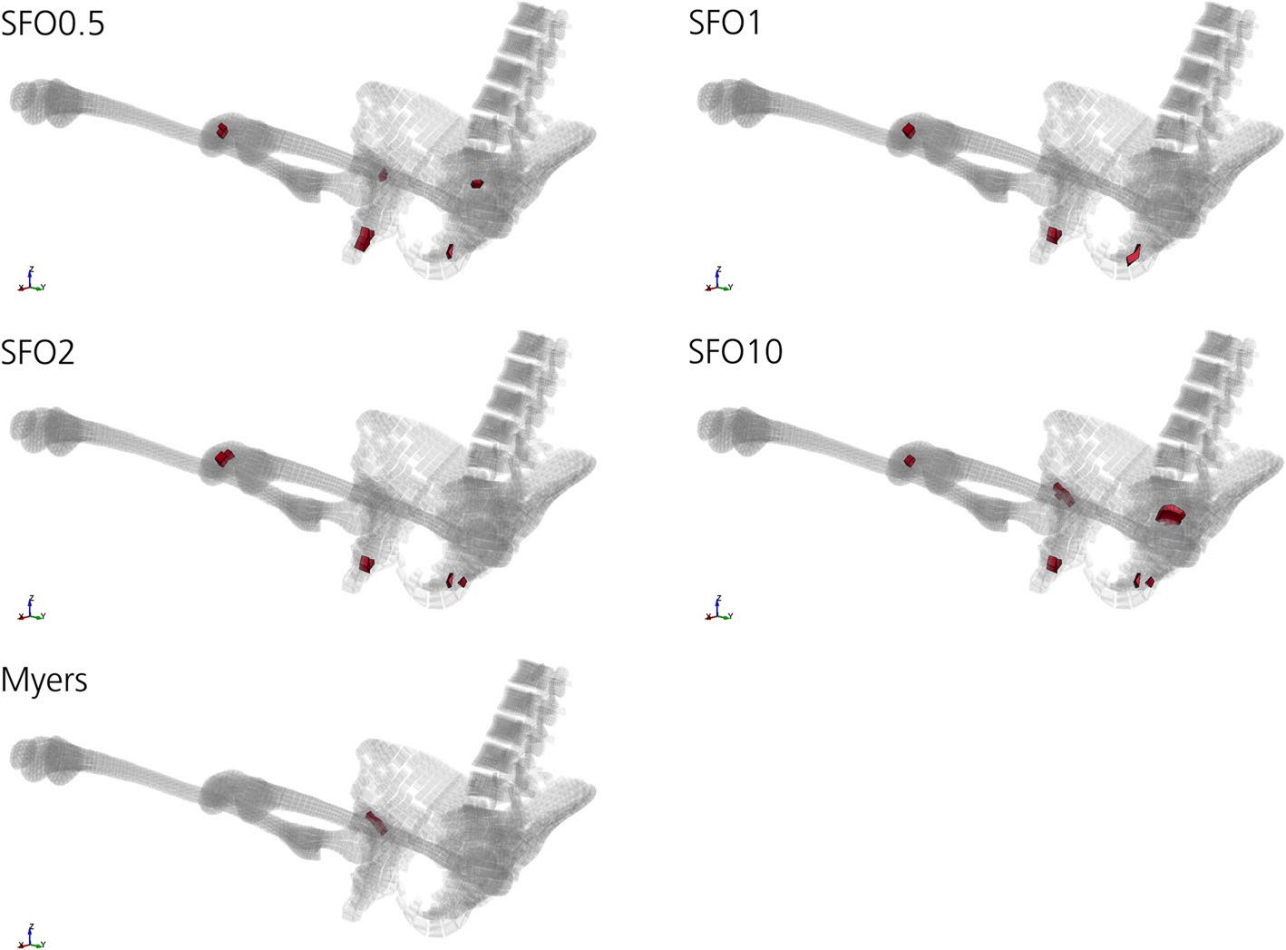
Spongy bone elements with 1^st^ principal strain greater than 1.5% over the entire computation time. The threshold was exceeded in the following body regions: right hipbone (all), left hipbone (all except Myers), left patella (all except Myers). Peak volumes of failed elements were 2.23 cm^3^ (SFO0.5, 75 ms), 1.65 cm^3^ (SFO1, 75 ms), 2.01 cm^3^ (SFO2, 70 ms), 6.53 cm^3^ (SFO10, 65 ms), 2.71 cm^3^ (Myers, 70 ms)

Data on first principal strains of spongy bones (Fig. 13) show an increase in predicted injury risk based on failed element volume in the following order: SFO1, SFO2, SFO0.5, Myers and SFO10. SFO10 had by far the highest failed element volume. Affected body regions were the right hipbone (all), left hipbone (all except Myers), left patella (all except Myers).

Therefore, no clear tendency between muscle stiffness changes and predicted injury risk of bones of the lower part of the body based on first principal strain evaluation (Figs. 12, 13) was found in this study.

#### Muscle and soft tissues

For muscle and soft tissues, effective strains were used for injury prediction, as well as the Cumulative Strain Damage Measure (CSDM) for different body regions and tissues based on effective strains (“Injury prediction”).

**Fig. 14:**
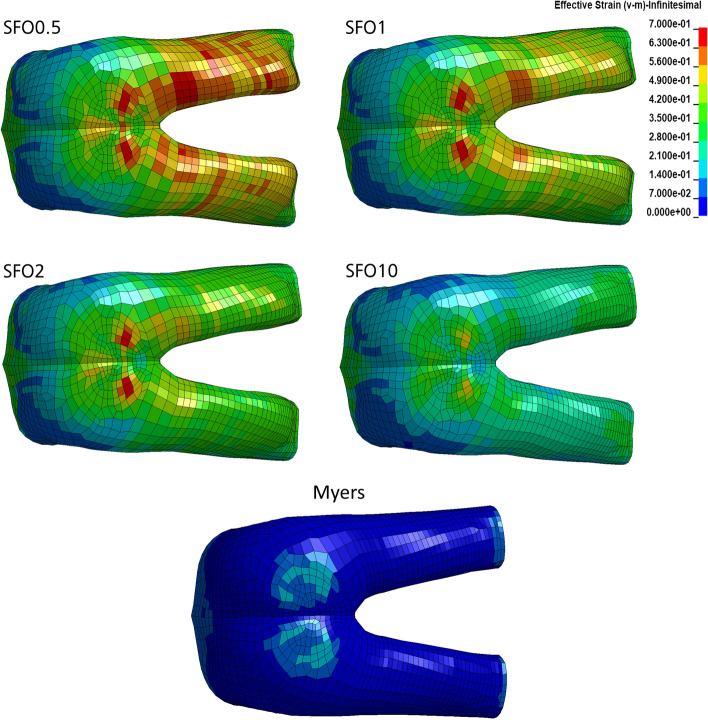
Effective strains in the muscle tissue of the pelvis and thigh after 75 ms computation time

**Fig. 15:**
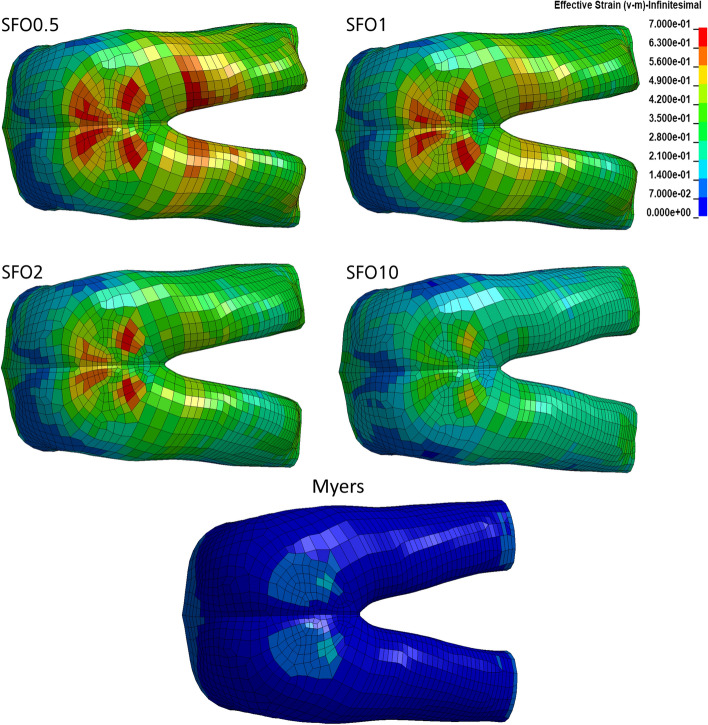
Effective strains in the soft tissue of the pelvis and thigh after 75 ms computation time

**Fig. 16:**
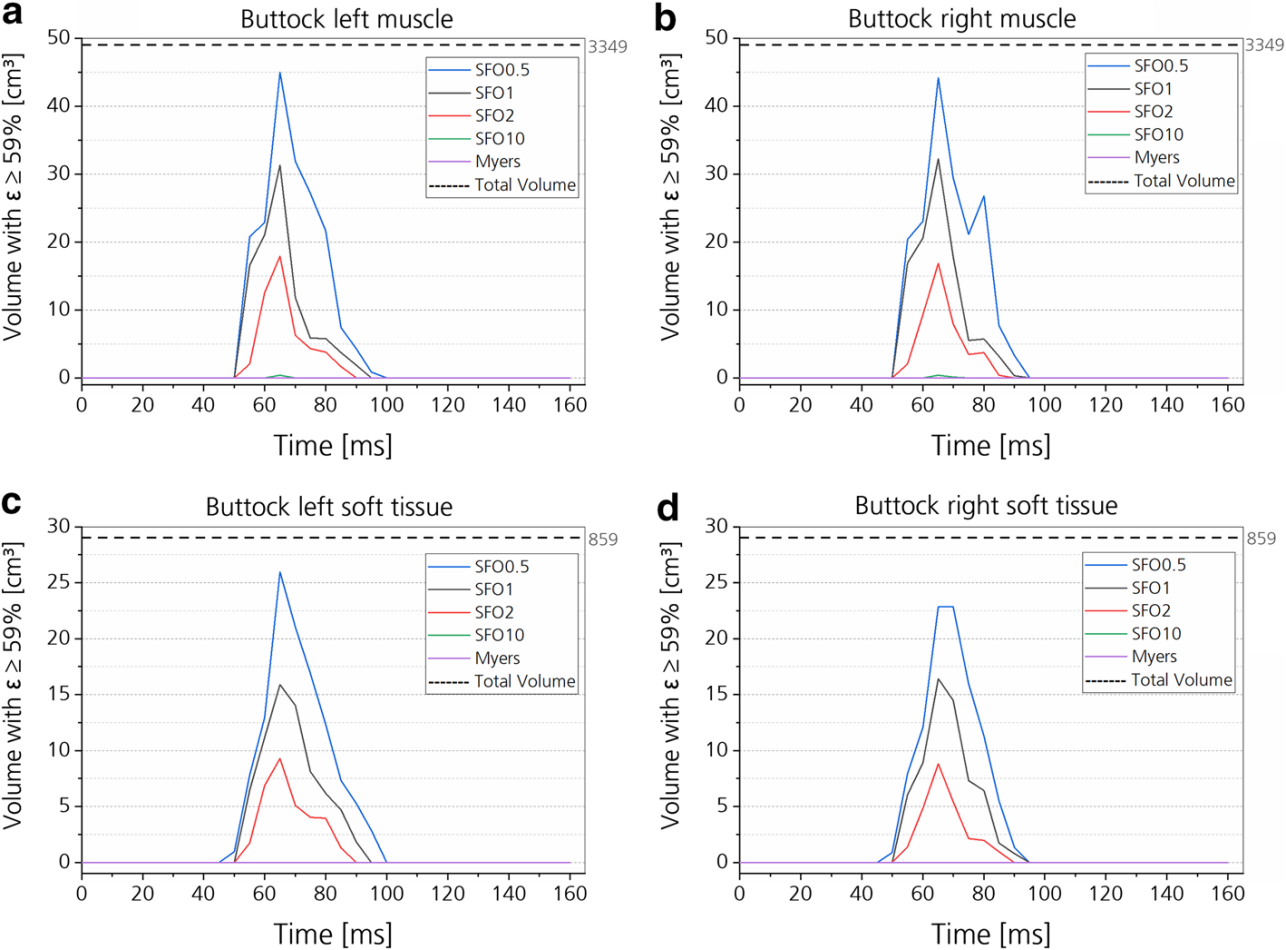
CSDM values of the buttock. Respective volume for each muscle stiffness state is plotted over time for muscle (**a**, **b**) and soft tissues (**c**, **d**), subdivided into the right and left side of the body

**Fig. 17:**
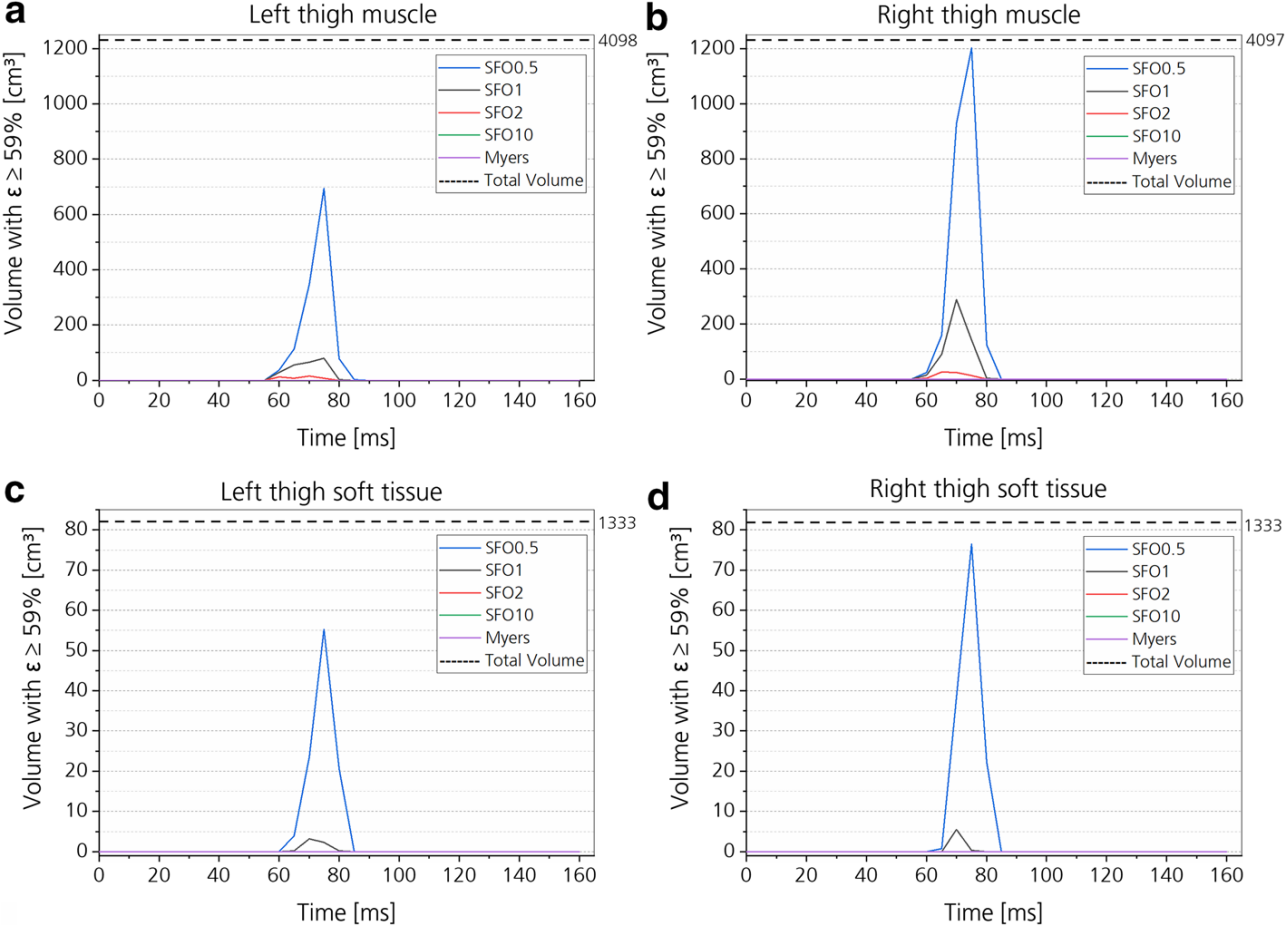
CSDM values of the thighs. Respective volume for each muscle stiffness state is plotted over time for muscle (**a**, **b**) and soft tissues (**c**, **d**), subdivided into the right and left side of the body

Effective strain color plots in the dorsal view are shown in Figs. 14 and 15. They were obtained after 75 ms, where for most regions, maximum loading was found. Highest strains in muscle and soft tissue were found in the periphery of the anus and in the dorsoproximal region of the thigh. In the buttock, higher strains were found for the soft tissue (outer layer) compared to the muscle tissue (inner layer). In contrast, high strains were distributed over a larger area for the thigh muscle tissue than for the soft tissue. Peak effective strain values were continuously decreasing with increasing muscle stiffness for all analyzed tissue types and body regions, which is described in detail below. The CSDM value (Figs. 16, 17) also decreases with increasing muscle stiffness for all analyzed tissue types and body regions. Therefore, it becomes evident that increasing muscle stiffness reduces the predicted muscle and soft tissue injury risk in frontal impact scenarios based on strain-dependent data.

Peaks in effective strain of muscle tissue (Fig. 14) for each stiffness case are: SFO0.5 = 103.9% (*t* = 70 ms); SFO1 = 95.3% (*t* = 70 ms); SFO2 = 80.4% (*t* = 65 ms); SFO10 = 61.2% (*t* = 65 ms); Myers = 30.2% (*t* = 75 ms). Data on 1st principal strain showed a similar distribution of peak strains, but lower maximum values. Peaks in 1st principal strain are: SFO0.5 = 82.1% (*t* = 60 ms); SFO1 = 80.5% (*t* = 60 ms); SFO2 = 78.6% (*t* = 60 ms); SFO10 = 64.4% (*t* = 65 ms); Myers = 20.5% (*t* = 85 ms).

Peaks in effective strain of soft tissue (Fig. 15) for each stiffness case are: SFO0.5 = 81.4% (*t* = 80 ms); SFO1 = 80.6% (*t* = 80 ms); SFO2 = 77.6% (*t* = 80 ms); SFO10 = 56% (*t* = 65 ms); Myers = 21.5% (*t* = 75 ms). Data on 1st principal strain showed a similar distribution of peak strains, but lower maximum values. Lower strain peaks are found for the periphery of the anus. Peaks in 1st principal strain are: SFO0.5 = 73:3% (*t* = 75 ms); SFO1 = 67% (*t* = 70 ms); SFO2 = 57.9% (t = 70 ms); SFO10 = 45% (t = 65 ms); Myers = 20.2% (*t* = 85 ms).

## Discussion

### Volumetric and muscle element stiffness

With increasing SFO values, the slope of the effective stress–strain curve is increasing for solid elements of the muscle tissue of the buttock (Fig. 4). This confirms the expected direct influence of the SFO scaling factor on the tissue stiffness, as the slope of the effective stress–strain curve is directly correlated with the element stiffness. As shown in Fig. 5, literature data on Young’s moduli of muscles increase with increasing voluntary muscle tension within the same studies (indicators), e.g., LEVINSON et al. [27] or SHINOHARA et al. [29]. Regarding this fact, data shown in Fig. 4 are in general agreement with literature data. For the highest material stiffness parameter (SFO10), the stiffness value obtained from simulation (Fig. 5, SFO10—green lines) was much higher than the corresponding data of contracted muscle from literature (Fig. 5, ‘contracted’) obtained by shearwave or sonoelastography measurement (Table 1), where usually only little strain of 0.1 to 2% is required for stiffness measurement [53]. However, other literature sources [25] performed experiments at much higher strain rates (1/s to 25/s) than the other sources of Fig. 5 and Table 1 (e.g., 0.4 s^−1^ [26]). They show a reverse trend by having much higher Young’s moduli for the relaxed (passive) than for the tensed (active) state. Muscle material properties are significantly strain-rate dependent in the passive muscle state (Fig. 5, [25, 54]). However, for the active muscle state [25], differences in material properties can only be found between quasi-static and dynamic strain rates (Fig. 5), while there is no significant difference within dynamic strain rates [25]. The data of the SFO10 case are in range of experimental data at dynamic strain rates [25] for the active state, while all SFO cases are much lower compared to the passive moduli (Fig. 5). If stress–strain data for different strain rates from experiment [25] are incorporated in the MAT_SIMPLIFIED_FOAM material model via a TABLE ID, results are in range of experimental data of tension tests regarding the Young’s moduli (Fig. 5, Myers—purple lines). Therefore, strain-rate dependency of muscle tissue should be considered when muscle behavior is analyzed and modeled in future approaches in a biofidelic way.

The large variety of experimental setups, muscle samples, measurement techniques and consequently Young’s moduli in literature currently makes it difficult to determine specific SFO values for each muscle in THUMS. Measurements at different strain rates for different human muscles, comparable to [25], would be necessary to further develop human body models, but are of course not possible due to ethical reasons. The motivation of this work was to cover a large area of possible material stiffnesses that resembles the stiffness of different contractile states of the literature data. For future optimization, this approach would benefit from consistent experimental data on all skeletal muscles relevant for occupant safety at different strain rates in the passive and active state.

### Tissue motion

As observed by Pain and Challis (2002) for a 27 year old volunteer [28], the intrasegmental tissue motion is 50% lower in a tensed arm than in a relaxed arm. Figure 10a shows a decrease in tissue motion with increasing muscle and soft tissue stiffness (SFO value). Therefore, the simulation data are generally consistent with literature data [28].

### Computation time and model stability

Based on the results, no significant effect of different material parameter values on the computation time was found. The reason for the small differences in computation time is expected to be based on server utilization and IO processes rather than changes in the material model parameters. Higher computation times were found when calculating the simulation on the server, while lower computation times were found for the isolated CPU in comparison to the default stiffness case (SFO1). For both, no significant change in computation time was found. Also, for the simplified cuboid model, no significant difference in computation time was found, supporting the argument above.

The model stability was analyzed based on whether or not the simulations reached the final termination cycle after 160 ms simulation time. The model stability was unaffected by the muscle stiffness changes, except for the SFO0.5 simulation, which ended with an error termination after 125 ms due to negative volume errors in the knee region. Therefore, it can be claimed that decreasing the muscle stiffness from the default value results in model instabilities. However, as the underlying material data used for SFO1 (default) were obtained from the PMHS test, it can be assumed that this should be the lowest material stiffness definition for human muscle tissues and that a lower stiffness would not be feasible.

Other material models from the default LS-DYNA library [11, 24] offer a variety of options to model the mechanical behavior of muscle in more detail than MAT_SIMPLIFIED_FOAM and consider, e.g., passive and active muscle behavior or the anisotropic properties of muscle. However, when used in automotive HBM applications, their influence on the computation time and model stability compared to default HBM material models is still unknown and might very well change, depending on the level of detail at which the microscopic and macroscopic muscle properties are incorporated. As already stated in former studies [7, 55], a trade-off needs to be found between biological accuracy on the one hand and calculation speed and data evaluability on the other. It is yet unknown, if a change in muscle material models could improve the THUMS behavior in terms of biofidelity, while maintaining reasonable computation time. This aspect should be considered in future studies in the field of occupant safety and muscle modeling.

### Injury prediction

Results from “Injury prediction” are discussed.

#### Bone tissues

According to literature, hip injuries are more frequent than knee or thigh injuries [34]. This can be confirmed based on the force- and strain-dependent injury prediction of this study (Figs. 10, 11, 12, 13).

The most frequently observed injuries due to frontal crashes are acetabular fractures [34], i.e., fractures in the hip joint. This is in compliance with the results regarding first principal strain of the cortical bones (Fig. 12). However, this cannot be confirmed using effective plastic strain-based injury prediction (Fig. 11). Although high loadings of the acetabulum were observed, the threshold of 1.5% effective plastic strain was not exceeded. Peaks were rather found for the right hipbone and the lumbar vertebrae.

The second most frequent fractures are found in the pubic ramus, sacrum and the femoral head and neck region. Respective data are listed in the Crash Injury Research and Engineering Network (CIREN) [34]. Although higher loading of some of these body regions was found, they were not regions with highest peak strain values (comp. Figs. 11, 12, 13). The reason for this might be that the high degree of simplification of the vehicle has a significant impact on the location of predicted injuries. Results regarding injury risk might therefore not be comparable to accident statistics, as the real vehicle interior and the simplified vehicle of this study differ too much. Further, other boundary conditions, such as vehicle impact speed and deceleration might differ in the accident statistic from the setup of this study, resulting in differences in predicted injury outcome and real injury outcome.

Based on the results of this study, muscle stiffness can affect the number and location of bone fractures, but not their general occurrence during frontal impact. A detailed numerical replication of real-world accidents regarding all boundary conditions using THUMS with different muscle stiffness states would be necessary, to determine which muscle stiffness states leads to a more realistic injury risk prediction.

##### Force-dependent injury prediction

Based on the force-dependent injury prediction, increasing muscle stiffness leads to a higher probability of hip fracture or dislocation (Fig. 10). In contrast, increasing the muscle stiffness in muscle and soft tissues surrounding the knee joint decreases the probability of knee injuries (Fig. 10, purple line).

Although the injury risk curve was developed for frontal impacts, such as the one simulated in this study, the setup shown in this study is not exactly matching the PMHS test setup from the experimental study, where the respective knee and hip injury risk curves were determined [52]. Further, the effect of the hands partly slipping off the steering wheel in some simulations cannot be exactly quantified. This should be addressed in future studies.

In an experiment where a medicine ball was dropped on the thigh of a volunteer [56], an increase of 11% in peak impact force from the relaxed to the voluntarily contracted muscle state was observed. Likewise, an increase in peak force with increasing muscle stiffness parameter value was found between the THUMS skin and the seat [56]. An increase by 11% of the peak force was determined, but only from the SFO1 to the SFO2 stiffness case. Therefore, the SFO2 stiffness value might be considered as the correct value to model partial voluntary muscle contraction of the human upper thigh. To verify this assumption regarding tissue motion [28] and peak impact force [56], the respective impact case should be replicated numerically.

##### Strain-dependent injury prediction

In general, hardly any elements of the THUMS cortical and spongy bones did exceed the respective threshold of 3% [57]. Higher impact velocities might be necessary to exceed the threshold. Therefore, a lower threshold of 1.5% was selected to allow a comparison of strain-dependent bone injury risk assessment.

Depending on the selected strain type, different impacts of the muscle stiffness changes on injury risk can be obtained for cortical bone. Effective plastic strain-based injury prediction (Fig. 11) shows a rather clear trend of increasing peak loading on the cortical bone with increasing muscle stiffness. First principal strain-based injury prediction shows an arbitrary correlation of muscle stiffness and peak loading for cortical bone (Fig. 12) and spongy bone (Fig. 13).

To confirm the results from one of the different strain values over the other, additional studies are necessary, where the injury risk could be determined for isolated body parts. A comparison with experimental studies would be essential for verification, although the effect of muscle stiffness on human bone injury risk can only be considered to a certain limit due to ethical reasons. Literature on experimental animal testing might rather be considered for validation of simplified simulation setups, such as Myers et al. [25].

##### Comparison: force- and strain-dependent injury prediction

Results from force- and strain-dependent bone injury prediction correlate to a certain extent. Similarities regarding the impact of muscle stiffness on bone injury can be found for the force-dependent injury probability of the hip (Fig. 10b) and effective plastic strain of the cortical bone (Fig. 11), as both suggest an increase in bone injury risk with increasing muscle stiffness. Results regarding first principal strains are not correlated with the force-based injury prediction.

#### Muscle and soft tissues

Increasing the muscle and soft tissue stiffness had a clear tendency of decreasing the injury risk in the simulation (Figs. 14, 15, 16, 17). In Figs. 16 and 17, effective strain was chosen as injury criterion with a value of 59%, representing the lower limit of the failure threshold of 95 ± 36% [58]. Therefore, the CSDM calculation might overestimate the muscle and soft tissue injuries. The effective strains and CSDM curves always decrease with increasing muscle stiffness. Only the SFO10 and Myers stiffness reduced the effective strain in the muscle and soft tissue to a value below the threshold of 59%.

## Outlook and limitations

Certain experimental studies that were compared to the frontal impact simulation results showed major differences regarding the test setup [28, 56, 58]. As the topic of muscle stiffness effects on occupant safety has not been investigated experimentally to the extent necessary for comparison with this study, the literature data presented are the closest approximation currently available in this field to the best knowledge of the authors. However, in future studies, the test setup mentioned above should be represented in numerical simulations using AHBM to further investigate muscle material properties and the relevance of muscle stiffness changes in a variety of loading scenarios.

The approach of this work, where muscle stiffness changes were predefined, was tested for THUMS V5 in this study. The same approach would also be applicable to other human body models, if MAT_SIMPLIFIED_FOAM is used for muscle and soft tissue modeling. A comparison between different material models regarding computation time and biofidelity would further be of interest for different muscle stiffness states in AHBM applications. Some of these models allow to define an activation curve dependent stiffness scaling. This would enable the user to define a functional dependency of the 1D and 3D muscle systems on the material card level, which would bring the AHBM muscle model closer to the biological role model.

This work showed that isometric contraction and the resulting change in muscle stiffness has an influence on the AHBM behavior and affects the predicted injury outcome. Therefore, muscle stiffness changes should be considered in different fields of biomechanics, such as the automotive sector, powered-two-wheeler safety, medical engineering, ergonomics or seat comfort analysis in the future.

## Conclusion

In this study, we presented an approach to consider isometric contraction of muscles and the resulting change in stiffness of muscle tissues for the human body model THUMS. Different stiffness states were predefined. As expected, the scaling factor of the engineering stress–strain curve (SFO value) can be used to scale the stiffness of volumetric muscle elements in THUMS. It was shown that stiffness changes in the buttock and pelvis region have an influence on the occupant kinematics and peak impact forces between THUMS and the simplified seat as well as on the loading and injury risk of bone, muscle and soft tissues of this body region. Although limitations regarding differences of the experimental setup from literature and the numerical setup exist, the results were in good agreement. Changes in muscle stiffness had no significant effect on the computation time and model stability. Server utilization and IO processes seemed to have a greater impact on the computation time than changes of the material model parameters. This was shown for different time-step sizes and hardware setups. Applicability of the approach of predefined muscle stiffness is given for research and industrial applications in terms of computational costs. In the future, comparative studies on the effect of different volumetric muscle material models in AHBM applications regarding biofidelity and computational cost would be of interest.

## Methods

### Simulation setup

**Fig. 18:**
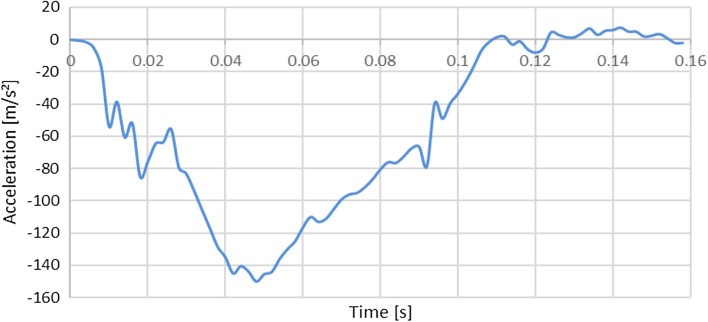
Acceleration curve used for the simplified crash scenario. As the vehicle is accelerated backwards, negative acceleration values are shown in this graph

The human body model THUMS, representing a 50 percentile average sized American adult male as an occupant, was used in this study. The braced muscle contraction state was predefined, during which the THUMS is pushing itself off the steering wheel and is stepping on the break. The most relevant vehicle parts (steering wheel, seatbelt, inflatable airbag, seat, footrest and kneebolster) were modeled as simplified and rigid, except for the airbag and seatbelt, see Fig. 20 in Appendix. The HBM was restrained by a seatbelt using both 1D seatbelt elements to include sliprings, pretensioner and retractor nodes efficiently and 2D shell elements for the contact to THUMS during the crash pulse. By accelerating the vehicle backwards according to a predefined acceleration curve (Fig. 18), a simplified frontal impact scenario is simulated. Occupant kinematics during impact are shown in Appendix (Fig. 20).

### Muscle stiffness parameters

**Fig. 19:**
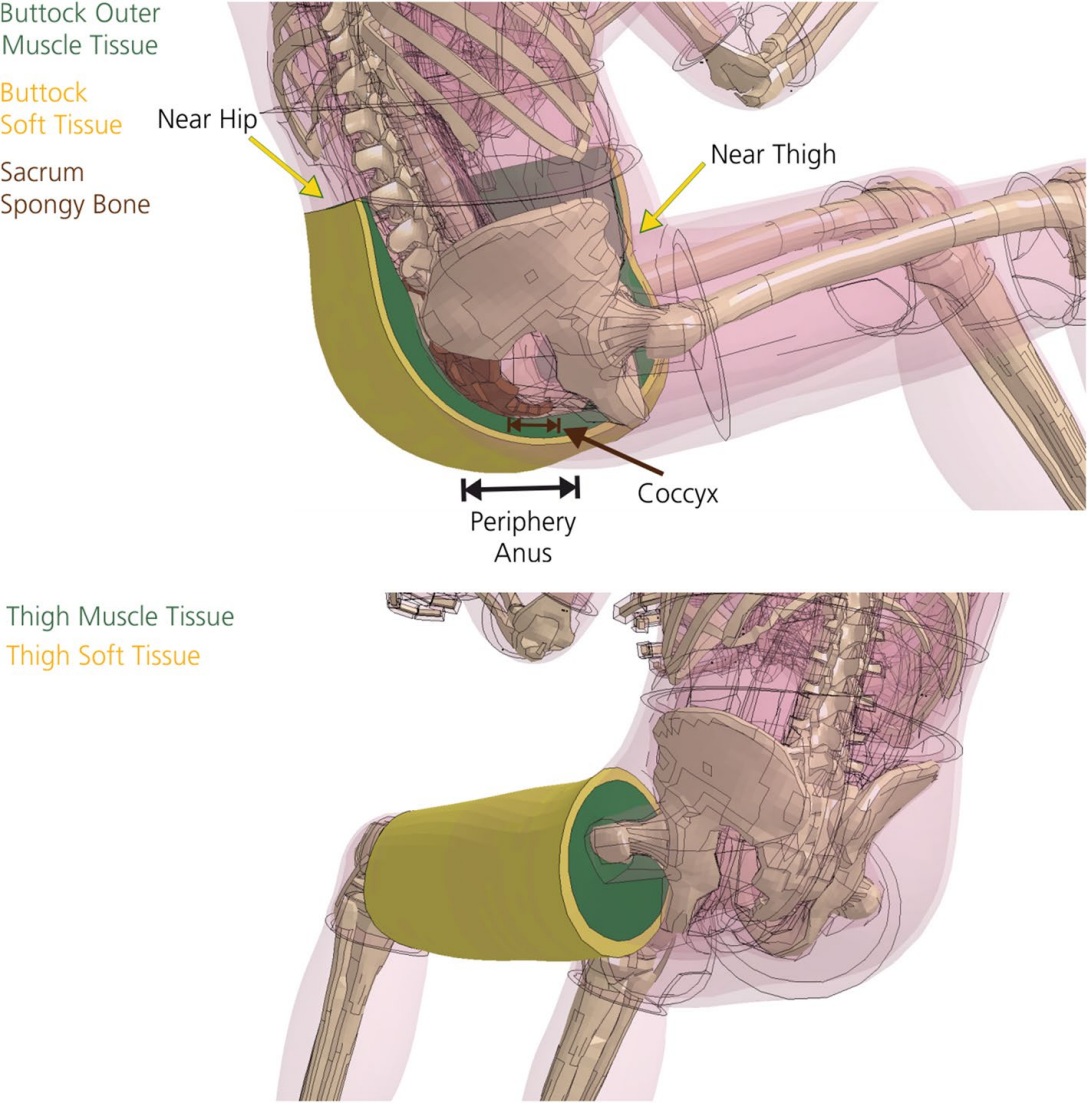
Analyzed body region: buttock (top) and thigh (bottom)

**Fig. 20:**
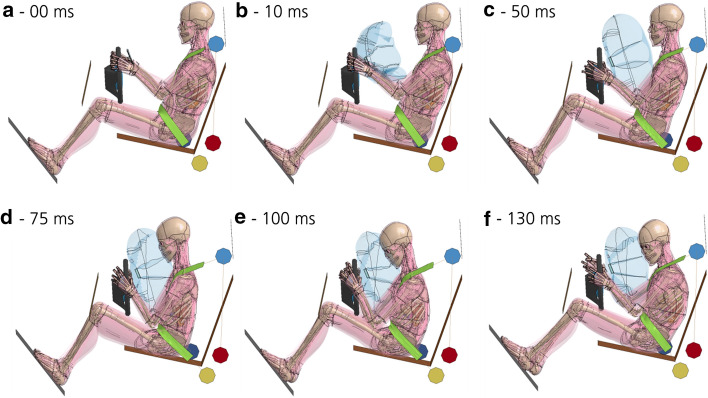
Simplified frontal crash pulse with THUMS. This graph is identical to Fig. 3

**Fig. 21:**
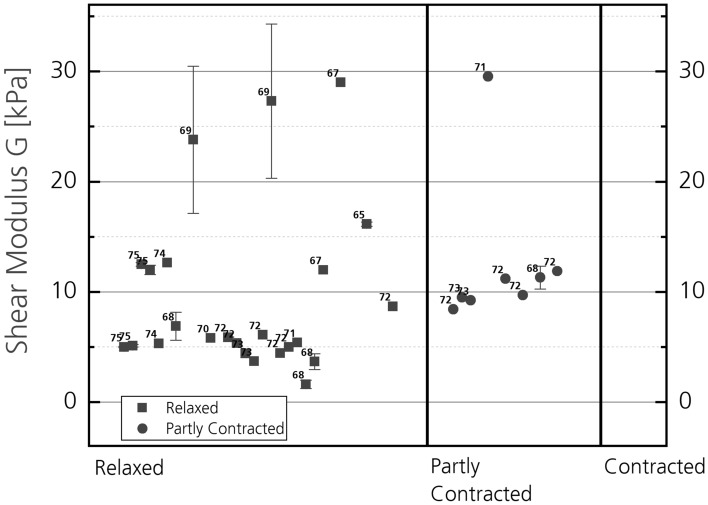
Literature data on shear moduli for different contractile states of different muscles (in vivo) or muscle samples (ex vivo) from humans, bovines and porcines. Indicators refer to the respective literature source, referenced and further explained in Table 1. Apart from the subdivision in different contractile states, there is no correlation on the abscissa of the data points. Distances of data points within one group are arbitrary

The material model is, among others, defined by the linear bulk modulus (KM), the shear modulus (G), an engineering stress–strain load curve (LC), a damping coefficient (MU) and a limit stress value (SIGF). The LC is only defined as such if the specimen dimensions are calibrated (equal to 1) under uniaxial loading. The variable which showed the most promising results in terms of stiffness alteration was the scaling factor of the ordinate value (SFO) that scales the engineering stress value of the load curve (LC). The default tissue stiffness and, accordingly, the default LC shape of MAT_SIMPLIFIED_FOAM in THUMS V5 was defined to represent overall PHMS behavior. The variety of scaling factors (SFO values) was selected in a way that a wide range of different muscle stiffness states is covered. The four SFO values were chosen as: (1) SFO = 0.5 for reduced stiffness, (2) SFO = 1.0 for default stiffness (THUMS V5, PMHS data [2, 14]), (3) SFO = 2.0 for slightly increased stiffness (possibly representing partial, voluntary contraction) and (4) SFO = 10.0 for highly increased stiffness (possibly representing maximum, tetanic contraction). The muscle stiffness was altered for muscle and soft tissue elements of the buttock and thigh that came into contact with the seat bottom (green and yellow parts in Fig. 19). This body region was analyzed and compared with literature data, mainly Pain and Challis and Tsui and Pain [28, 56]. To determine the effect of the SFO value on the material stiffness and for comparison with literature data, the effective stresses and effective strains were analyzed. Additionally, a fifth stiffness case was defined, where strain-rate-dependent data were incorporated in the model (Myers) via a Table ID using literature data obtained from experiments with rabbit muscles [25]. This Myers stiffness case was incorporated in all muscle and soft tissue elements of THUMS, not only for the buttock and thigh.

The effective stress, also known as von Mises stress, is defined as:

10 $$\sigma_{{{\text{effective}}}} = \frac{1}{\sqrt 2 } \sqrt {\left( {\sigma_{1} - \sigma_{2} } \right)^{2} + \left( {\sigma_{1} - \sigma_{3} } \right)^{2} + \left( {\sigma_{2} - \sigma_{3} } \right)} ,$$

where ${{{\sigma}}}_{1}$, ${{{\sigma}}}_{2}$ and ${{{\sigma}}}_{3}$ are the principal stresses. The effective strain, expressed in tensorial notation, is defined as:

11 $$\varepsilon_{{{\text{effective}}}} = { }\sqrt {\frac{2}{3}\varepsilon_{ij} \varepsilon_{ij} } .$$

### Simulation environment and computation time

The equation of conservation of momentum (subsection “Conservation equations and finite element method”, (1)) has to be solved numerically with constitutive models using the explicit time integration method for the loading scenario described above. By application of the explicit FE code MPP LS-DYNA v971 revision 7.1.2_95028 single precision (LSTC), the numerical solution was calculated using 16 cores on a Supermicro Computer (3.20 Ghz Intel® Xeon® CPU E5-1680 v3 processors with 128 GB RAM using Melanox Infiniband) running on CentOS Linux release 7. To achieve reasonable computation times, the time-step size for mass-scaled solutions in the LS-DYNA CONTROL_TIMESTEP [59] keyword was set to 0.001 ms by selective mass scaling^2^ for all simulations.

To analyze the impact of muscle stiffness on the computation time, different keyword and hardware setups were compared. On the one hand, the dt2ms value of the CONTROL_TIMESTEP was set to -0.001, thus fulfilling the CFL criterion by mass scaling. For all dt2ms = − 0.001 simulations, the mass of 85.29 kg of the entire simulation setup increased by 2.67% (2.28 kg). This was the standard setup for the simulation of this work, used for analysis of the injury outcome, tissue motion and peak impact force and the material behavior. On the other hand, dt2ms was left blank, thus achieving the CFL criterion by altering the minimum time-step size in dependence of the smallest element size. Regarding hardware setups, simulations were calculated on the server, where other users could perform simulations at the same time, using the setup mentioned above with 16 cores. For comparison, simulations were also calculated on an isolated CPU with 8 physical cores without hyperthreading one after another. Besides the four SFO stiffness simulations, the SFO10 value and strain-rate-dependent data (Myers) were applied to all volumetric MAT_SIMPLIFIED_FOAM muscle and soft tissue parts to generate ‘worst case scenarios’ regarding computation time.

The influence on computation time was further analyzed in simplified simulations, where a cube model consisting of eight cuboid solid elements (edge length: 1 mm) modeled with the MAT_SIMPLIFIED_FOAM model from THUMS buttock and thigh muscle and soft tissues. No loading was applied. The simulation ran on the isolated CPU for 50 ms with no defined time-step size.

The influence of muscle stiffness on the THUMS model stability was analyzed based on whether or not the simulation reached the final calculation cycle of explicit time integration for mass-scaled solutions (dt2ms). The termination time, meaning the predefined simulation time after which the simulation automatically stops, was set to 160 ms.

### Injury prediction

The use of AHBM offers a variety of injury criteria that can be considered to determine the injury risk. Strain-based and force-based injury risk prediction are among the most commonly established criteria to determine injury risk of bone, muscle and soft tissue using HBM based on current research on injury risk assessment [52, 60].

Using recent injury risk curves for the knee, distal femur and hip developed for use in frontal impacts [52], the peak force value acting on the knee and hip can be used to determine the probability of hip fractures and dislocation as well as patella and distal femur fractures. The effect of muscle stiffness changes is analyzed for peak force values of the knee and hip. For the hip, contact forces between the THUMS skin and the rigid seat bottom were used to determine injury probability, while for the knee, contact forces between the THUMS skin and the knee bolster were determined.

According to literature, the fracture limit strain value for bone is 3% [57]. Wolfram and Schwiedrzik (2016) list several sources that cover similar ultimate strain values for cortical bone [61]. For spongy bone, ultimate strain values vary between 1 and 11% [62, 63], depending on, e.g., the donors’ health conditions (osteoporosis) and the loading type (tension, bending, compression).

Bone injuries were predicted based on the accumulation of effective plastic strain (over the entire crash simulation time $t$) exceeding a certain threshold. The effective plastic strain ${\varepsilon }_{\text{eff}}^{p}$ is defined as:

12 $$\varepsilon_{{{\text{eff}}}}^{p} = \mathop \smallint \limits_{0}^{t} \sqrt {\frac{2}{3}{\text{d}}\varepsilon_{ij}^{p} {\text{d}}\varepsilon_{ij}^{p} } ,$$

with the plastic strain ${\varepsilon }^{p}$. Further, first principal strains were analyzed. As the common limit value of 3% of cortical bone was hardly exceeded by any bone elements, a lower threshold of 1.5% was chosen for both cortical and spongy bone elements to allow a comparison of muscle stiffness effects on injury risk and a comparison to accident statistics and injury distribution from literature.

As observed in tensile tests with human muscle tissue connected to the native bone [58], the maximum elongation of the muscle sample reaches 95 ± 36%. For muscle and soft tissue injury prediction, maximum effective strains were found in both tissues of THUMS at different predefined stiffnesses, and are analyzed and compared. Effective strain distributions of the outer surface of respective tissues are compared. Further, the CSDM, commonly used for brain injury prediction [64], was calculated, accumulating all elements exceeding the lower limit of the maximum elongation (59%) mentioned above. This allows the comparison of effective strain development for whole muscle and soft tissue body parts over the entire computation time for the different stiffness cases.

## Data Availability

Due to a license agreement with Toyota Motor Corporation and Toyota Central R&D Labs., Inc. regarding the THUMS™ AM50%ile Version 5.01 human body model, certain limitations are given regarding data availability. Data originating from simulation in LS-DYNA and visualization in LS-PrePost (©2011–2019 LSTC) can only be reproduced if the respective owner has access to the THUMS Version 5.01 model mentioned above. Further, material data of the seatbelt are not publically available. Apart from that, the datasets used and/or analyzed during the current study are available from the corresponding author on reasonable request.

## Appendix

### General overview of the crash simulation

The general setup and progress of the simplified frontal impact simulation using THUMS and a simplified vehicle model are shown in Fig. 20 for the SFO1 case regarding muscle and soft tissue stiffness. Maximum stresses and maximum strains were found after 60–80 ms for the observed volumetric muscle, soft tissue and bone elements. The airbag fully inflates within 25 ms. The head of THUMS hits the airbag after 60 ms. The knees hit the kneebolster after 45 ms. The buttock hits the bottom seat after 30 ms. The maximum resultant force between THUMS and the seat belt was found at 70 ms. After 95 ms, the THUMS gains negative momentum and moves in direction of the seat back, partly due to repulsive forces of vehicle parts resulting from the impact and because the seatbelt still retains the THUMS to the seat. First pre-tensioning of the seatbelt occurs from 0 to 20 ms, where the belt comes into contact with the THUMS skin. This is followed by an extension phase of the seatbelt. The THUMS moves into direction of the airbag during impact, where the seatbelt follows its movement by extension. This occurs, like the pre-tensioning, by movement of 1D seatbelt elements through the different sliprings (blue, dark blue). For pre-tensioning, the retractor (red) pulls in 1D seatbelt elements in its proximity, while extension is realized by releasing elements from the retractor, which were earlier pulled in during pre-tensioning. A second seatbelt tensioning phase is initiated after 110 ms, as 1D seatbelt elements are again pulled in by the retractor.

### Literature data of muscle tissue stiffness measured using elasticity imaging

Figure 21 contains supplementary data on Shear Moduli *G* besides the data on Young’s moduli *E* from Fig. 5. Detailed information, including the type of samples and measurement technology are listed in Table 1. Many of the literature sources presented were previously summarized by Sarvazyan et al. [40].

**Table 1:** Data about the elastic and viscoelastic properties of different muscle tissues

Source	Elastic properties (kPa)	Viscosity (Pa * s)	Sample	Measurement technology
Basford et al. (2002) [65]	**Shear modulus G** **16.16 ± 00.19 kPa** (tissue stiffness defined as equal to G in study)	No data	*Musculus gastrocnemius* (human)	Magnetic Resonance Elastography (MRE)
Chen et al. (1996) [66]	**Young’s modulus** **2.12 ± 0.91 kPa** (ultrasound) **1.53 ± 0.31 kPa** (Instron)	No data	*Musculus longissimus* (bovine)	Ultrasound and Instron methods
Chen et al. (2009) [67]	**29 kPa** (along the muscle fiber) **12 kPa** (across the muscle fiber)	**9.9 Pa*s** (along fiber) **5.7 Pa*s** (across fiber)	Striated muscle fibers [in vitro] (bovine)	Shearwave dispersion ultrasound vibrometry (SDUV)
Debernard et al. (2013) [68]	**Shear modulus G** **3.67 ± 0.71 kPa** (VM, passive) **11.29 ± 1.04 kPa** (VM, 20% activity) **6.89 ± 1.27 kPa** (SR, passive) **1.61 ± 0.37 kPa** (adipose)	**4.5 ± 1.64 Pa*s** (VM, passive) **12.14 ± 1.47 Pa*s** (VM, 20% activity) **6.63 ± 1.27 Pa*s** (SR, passive)	*Musculus vastus medialis* (VM) *Musculus sartorius* (SR) Subcutaneous (connective) and adipose tissue	Multifrequency magnetic resonance elastography (MMRE)
Dresner et al. (2001) [69]	**Shear stiffness G** **23.8 ± 6.68 kPa** (bovine) **Ø 27.3 kPa** (**range: 8 – 34 kPa**) (human)	No data	Muscle tissue (ex vivo) (bovine) *Musculus biceps brachii* (human)	MRE
Eby et al. (2013) [70]	**Shear modulus G** **5.81 kPa** (at 90° elbow angle)	No data	*Musculus brachialis* (porcine, whole muscle specimen)	Shear wave elastography (SWE)
Gennisson et al. (2010) [71] (referenced by Eby et al. (2013) [70])	**5.4 kPa** (at 90° elbow angle) **29.54 kPa** (at 165° elbow angle)	No data	*Musculus biceps* (human)	Noninvasive supersonic shear imaging technique
Hoyt et al. (2008) [72]	**Shear modulus G** **5.87 kPa** (relaxed, RF, V1) **11.17 kPa** (contracted, RF, V1) **5.33 kPa** (relaxed, RF, V2) **9.70 kPa** (contracted, RF, V2) **6.09 kPa** (relaxed, BB, V1) **8.42 kPa** (contracted, BB, V1) **8.68 kPa** (relaxed, BB, V2) **11.88 kPa** (contracted, BB, V2) **4.45 kPa** (BF, V1) **4.98 kPa** (MG, V1)	**9.14 Pa*s** (relaxed, RF, V1) **11.88 Pa*s** (contracted, RF, V1) **9.72 Pa*s** (relaxed, RF, V2) **11.60 Pa*s** (contracted, RF, V2) **10.55 Pa*s** (relaxed, BB, V1) **11.90 Pa*s** (contracted, BB, V1) **9.73 Pa*s** (relaxed, BB, V2) **13.22 Pa*s** (contracted, BB, V2) **9.13 Pa*s** (BF, V1) **9.26 Pa*s** (MG, V1)	*Musculus rectus femoris* (RF) *Musculus biceps femoris* (BF) *Musculus gastrocnemius* (MG) *Musculus biceps brachii* (BB) (human) Two volunteers (V1, V2)	Sonoelastography
Myers et al. (1998) [25]	**Young’s modulus E** **1750 ± 1180 kPa** (passive, 1/s) **2450 ± 800 kPa** (passive, 10/s) **2790 ± 670 kPa** (passive, 25/s) **970 ± 340 MPa** (active, average strain rates)	No data	*Musculus tibialis anterior (New Zealand white rabbit)* *Active:* 19.3 N, nerve excitation, resulting tetanic true stress level	1750 Actuator displacement measured via linear variable differential transformer Optical data recorded
Krouskop et al. (1987)[26]	**Young ‘s modulus** **6.21 ± 0.48 kPa** (relaxed) **35.85 ± 1.38 kPa** (mild, supporting 2.26 kg weight) **108.94 ± 2.07 kPa** (maximum)	No data	(human adult missing his lower right leg, from above knee) Six volunteers Measurement at the femur *M. vastus* intermedius/*M. rectus* femoris	Doppler ultrasonic system and Instron
Levinson et al. (1995)[27]	**Young’s modulus** 30 Hz measurement: **79 ± 29 kPa** **103 ± 26 kPa 126 ± 26 kPa** For corresponding loads of 0 kg, 7.5 kg and 15 kg 60 Hz measurement: **25 ± 6.75 kPa** **75 ± 61 kPa 127 ± 65 kPa** For corresponding loads of 0 kg, 7.5 kg and 15 kg	Unable to quantify viscosity	*Musculus quadriceps femoris* Ten volunteers 30 Hz measurement: (human)	Sonoelastography
Ringleb et al. (2007) [73]	**Shear stiffness** **3.7 kPa** (1D) and **4.4 kPa** (2D) (relaxed) **9.5 kPa** (1D) and **9.22 kPa** (2D) (20% of maximum voluntary contraction)	No data	*Musculus vastus medialis* Five volunteers (human)	MRE correlated to electromyographic data 1D and 2D measurement techniques
Shinohara et al. (2010) [29]	**Young’s modulus** **40.6 ± 1.0 kPa** (relaxed) **258.1 ± 15.0 kPa **(30% voluntary contraction) **16.5 ± 1.0 kPa **(relaxed) **225.4 ± 41.0 kPa **30% voluntary contraction) **14.5 ± 2.0 kPa **(relaxed) **55.0 ± 5.0 kPa **(30% voluntary contraction)	No data	Human volunteer (age 42) *Musculus tibialis anterior* *Musculus gastrocnemius* *Musculus soleus*	Ultrasound shear wave imaging
Urban and Greenleaf (2009) [74]	**Shear elasticity** **12.65 kPa** (along the fiber) **5.32 kPa** (across the fiber)	**Shear viscosity** **2.91 Pa*s** (along the fiber) **1.05 Pa*s** (across the fiber)	Muscle fibers of muscle tissue (Porcine, ex vivo)	Ultrasonic pulse-echo method Tonebursts of 3.0 MHz with lengths of *T*_*b*_ = 200 µs repeated at a rate of 100 Hz
Urban et al. (2009) [75]	**Shear elasticity** **11.98 ± 0.43 kPa** (200 µs) **12.50 ± 0.17 kPa** (400 µs) (along fibers) **5.11 ± 0.11 kPa** (200 µs) **4.99 ± 0.06 kPa** (400 µs) (across fibers)	**Shear viscosity** **3.51 ± 0.21 Pa*s** (200 µs) **2.92 ± 0.09 Pa*s** (400 µs) (along fibers) **1.26 ± 0.11 Pa*s** (200 µs) **1.57 ± 0.05 Pa*s** (400 µs) (across fibers)	Muscle fibers of muscle tissue (porcine, ex vivo)	Ultrasonic pulse-echo method Tonebursts of 3.0 MHz with lengths of *T*_*b*_ = 200 µs and *T*_*b*_ = 400 µs repeated at a rate of 100 Hz

A variety of measurement technologies that can all be assigned to the field of ‘Elasticity Imaging’ or ‘Elastography’ [16] were used to obtain this data. Data were collected and assembled from publications listed in the column ‘Source’ by the author of this work

## Abbreviations

1D muscles: One-dimensional Hill-type muscle truss elements with passive and active properties to generate force at the joints via activation curves enabling musculoskeletal movement of human body models and to alter joint stiffness
3D muscles: Three-dimensional volumetric or solid finite elements used for modeling muscle tissues in human body models. These solid elements define the volumetric shape of biological muscles and are relevant for deformation due to external loads
AHBM: Active human body model
CFL: Courant–Friedrichs–Lewy criterion
CPU: Central processing unit
CSDM: Cumulative strain damage measure
Cycle: Cycle refers to one cycle of the explicit time integration, where displacements, velocities, accelerations of each node and stresses and strains of each element are calculated. The time step size, meaning the time increment to the next point of time, is calculated from the smallest element length using the CFL criterion. If a time step size is predefined, then the correct solution is reached via mass scaling
FEM: Finite element method
HBM: Human body model
Myers: Abbreviation of a simulation where higher muscle stiffnesses were defined based on the respective literature source containing strain-rate-dependent stress-strain curves of muscle tissues. These curves were used to define material properties of volumetric muscle and soft tissues of THUMS
PMHS: Post mortem human subject(s)
SFO: Scaling Factor of the Ordinate value. In this case, the ordinate value is the stress value of the effective stress-strain input curve of the volumetric muscle and soft tissue model of THUMS
THUMS: Total HUman Model for Safety
THUMS V5: THUMS Version 5. One of the latest AHBM of the Toyota Motor Corporation, which includes 256 contractible muscle truss elements enabling movement and altering the joint stiffness

## Footnotes

1 In this work, the *Total HUman Model for Saftey* (THUMS™) Version 5.01 was used. For readability, the abbreviation ‘THUMS’ is used in the following. If other THUMS models are referenced, the respective model versions are provided.

2 The abbrevation ‚- 0.001 ‘ will be used in the following as abbreviation for a ‘predefined time step of 0.001 ms achieved by selective mass scaling’, according to the usage in the LS-DYNA manual [59].
